# Spatiotemporal characterization of cellular tau pathology in the human locus coeruleus–pericoerulear complex by three-dimensional imaging

**DOI:** 10.1007/s00401-022-02477-6

**Published:** 2022-08-30

**Authors:** Abris Gilvesy, Evelina Husen, Zsofia Magloczky, Orsolya Mihaly, Tibor Hortobágyi, Shigeaki Kanatani, Helmut Heinsen, Nicolas Renier, Tomas Hökfelt, Jan Mulder, Mathias Uhlen, Gabor G. Kovacs, Csaba Adori

**Affiliations:** 1 Department of Neuroscience, Karolinska Institutet, Solnavägen 9, 17177 Stockholm, Sweden; 2 McGill University, Montreal, QC H3A 0G4 Canada; 3 Human Brain Research Laboratory, Institute of Experimental Medicine, ELKH, Budapest, Hungary; 4 Department of Pathology, St. Borbála Hospital, Tatabánya, Hungary; 5 Department of Neurology, Faculty of Medicine, University of Debrecen, Debrecen, Hungary; 6 Department of Old Age Psychiatry, Institute of Psychiatry Psychology and Neuroscience, King’s College London, London, UK; 7 Centre for Age-Related Medicine, SESAM, Stavanger University Hospital, Stavanger, Norway; 8 Institute of Neuropathology, University of Zurich, Zurich, Switzerland; 9 Department of Medical Biochemistry and Biophysics, Karolinska Institutet, 17177 Stockholm, Sweden; 10 Clinic of Psychiatry and Institute of Forensic Pathology, University of Würzburg, 97080 Würzburg, Germany; 11 LIM-44, University of Sao Paulo Medical School, Sao Paulo, Brazil; 12 Sorbonne Université, Paris Brain Institute-ICM, INSERM, CNRS, AP-HP, Hôpital de la Pitié Salpêtrière, 75013 Paris, France; 13 Science for Life Laboratory, Royal Institute of Technology, 10691 Stockholm, Sweden; 14 Tanz Centre for Research in Neurodegenerative Disease and Department of Laboratory Medicine and Pathobiology, University of Toronto, Toronto, ON Canada; 15 Laboratory Medicine Program and Krembil Brain Institute, University Health Network, Toronto, ON Canada

**Keywords:** Locus coeruleus, Tau pathology, Alzheimer’s disease, Three-dimensional, iDISCO, light sheet fluorescence microscopy

## Abstract

Tau pathology of the noradrenergic locus coeruleus (LC) is a hallmark of several age-related neurodegenerative disorders, including Alzheimer’s disease. However, a comprehensive neuropathological examination of the LC is difficult due to its small size and rod-like shape. To investigate the LC cytoarchitecture and tau cytoskeletal pathology in relation to possible propagation patterns of disease-associated tau in an unprecedented large-scale three-dimensional view, we utilized volume immunostaining and optical clearing technology combined with light sheet fluorescence microscopy. We examined AT8^+^ pathological tau in the LC/pericoerulear region of 20 brains from Braak neurofibrillary tangle (NFT) stage 0–6. We demonstrate an intriguing morphological complexity and heterogeneity of AT8^+^ cellular structures in the LC, representing various intracellular stages of NFT maturation and their diverse transition forms. We describe novel morphologies of neuronal tau pathology such as AT8^+^ cells with fine filamentous somatic protrusions or with disintegrating soma. We show that gradual dendritic atrophy is the first morphological sign of the degeneration of tangle-bearing neurons, even preceding axonal lesions. Interestingly, irrespective of the Braak NFT stage, tau pathology is more advanced in the dorsal LC that preferentially projects to vulnerable forebrain regions in Alzheimer’s disease, like the hippocampus or neocortical areas, compared to the ventral LC projecting to the cerebellum and medulla. Moreover, already in the precortical Braak 0 stage, 3D analysis reveals clustering tendency and dendro-dendritic close appositions of AT8^+^ LC neurons, AT8^+^ long axons of NFT-bearing cells that join the ascending dorsal noradrenergic bundle after leaving the LC, as well as AT8^+^ processes of NFT-bearing LC neurons that target the 4th ventricle wall. Our study suggests that the unique cytoarchitecture, comprised of a densely packed and dendritically extensively interconnected neuronal network with long projections, makes the human LC to be an ideal anatomical template for early accumulation and trans-neuronal spreading of hyperphosphorylated tau.

**Supplementary Information:**

The online version contains supplementary material available at 10.1007/s00401-022-02477-6.

## Introduction

The noradrenergic nucleus locus coeruleus (LC) is a key hub of the mammalian brain, connecting to virtually all parts of the central nervous system [12, 40, 45, 91, 98]. In humans, LC harbours around 22-52.000 pigmented neurons [16, 43], forming an elongated, rod-like bilateral structure in the dorsolateral pontine tegmentum [73]. LC is a major wakefulness-promoting nucleus and has a pivotal role in selective attention, motivations, anxiety, emotional learning, as well as in various autonomic functions like nociception and postural muscle tone [38, 75, 96, 98].

Yet, LC is a ‘*locus minoris resistantiae*’ in several neurodegenerative disorders, including Alzheimer’s disease (AD) [68, 69, 98], where LC neuronal loss is, in fact, more pronounced than the loss of cholinergic neurons in the nucleus basalis of Meynert [102]. Several prodromal symptoms of AD (like sleep disturbances, anxiety and depression) are consistent with LC dysfunction [75].

Pathological tau aggregation in the LC [28] in several tauopathies, including AD [21], progressive supranuclear palsy [54] or frontotemporal dementia [70] have been reported. The discovery of early tau cytoskeletal pathology in the LC of many subjects without any detected cortical tau lesions [23, 36, 88] led to a revision of the original Braak staging of NFTs [21], to include a precortical ‘stage 0’ [26]. Moreover, Braak and colleagues raised the hypothesis that LC is the starting point of AD tau pathology and that hyperphosphorylated tau spreads to the (trans)entorhinal cortex via noradrenergic (NA) LC axons [24]. However, this concept has recently been debated [48, 55, 56].

The distribution of tau cytoskeletal pathology, unlike Aβ dissemination, shows a correlation with the severity of cognitive impairments, and it is associated with clinical AD progression [87]. Thus, a deeper understanding of the origin, distribution and progression of tau pathology throughout the brain, including also the key hub LC, is highly relevant for the development of novel strategies to ameliorate cognitive changes associated with AD [47]. However, in vivo tau tracing is currently not possible in the LC due to its small size and the off-target binding of currently used tau PET radiotracers to neuromelanin [53]. The comprehensive post-mortem analysis of LC tau pathology in thin coronal sections used in the diagnostic routine is also challenging due to the small size and rostro-caudally elongated shape of the nucleus. At the same time, LC is an ideal target for the novel state-of-the-art 3D (volume) imaging technologies.

Volume imaging affords a comprehensive tissue overview of several millimeter ranges with cellular resolution, providing topological information in all three dimensions, as well as allowing detection of sparsely distributed cells or rare pathological alterations. Despite of the fact that volume imaging is in the frontline of system neuroscience today [95], studies with tissue optical clearing and large-scale volume imaging with light sheet fluorescence microscopy (LSFM) in human samples are still sparse in the field of neurodegeneration, mainly focusing on Aβ detection in cortical regions [41, 61], for review see: [71].

The objective of the present study is to explore the cytoarchitecture and tau cytoskeletal pathology in the human LC and adjacent pericoerulear (PC) structures. In particular, we aimed to analyze the spatiotemporal distribution of tau pathology on the cellular and regional levels, and we addressed the key issue of a possible early propagation and spreading of tau pathology within and from the LC. For this, we have applied the iDISCO + volume immunostaining and clearing technology [78], which allows a comprehensive three-dimensional visualization and quantitative spatial analysis of the LC/PC complex, in contrast to earlier studies with 2D imaging techniques that provided only a limited sectional view.

This approach enabled us to investigate the full spectrum of pathological tau positive cellular structures and to identify the earliest morphological signs of degeneration of NFT-bearing neurons. The large-scale spatial analysis also enabled us to track long axons of NFT-bearing cells that leave the LC, as well as to track processes of NFT-bearing LC neurons that target the 4th ventricle wall. Moreover, the full 3D analysis allowed us to capture the spatial distribution of pathological tau in distinct segments of the LC with preferential projections to different brain regions. Finally, nearest neighbour and in-depth cluster analyses quantitatively characterized the 3D topography of NFT-bearing cells, with implications for possible spreading routes of pathological tau at the level of individual neurons.

## Materials and methods

### Human brain samples

Human brains (Supplementary table 1, online resource) were obtained from the Department of Pathology, Szent Borbála Hospital, Tatabánya, Hungary (cases 1–4, 8–16 and 19), and from the Medical University of Vienna, Vienna, Austria (cases 5–7, 17–18 and 20). All procedures were approved by the Regional and Institutional Committee of Science and Research Ethics (ETT TUKEB 31443/2011/EKU, 518/PI/11, Hungary, renewed as: ETT TUKEB 15032/2019/EKU; Ethik-Kommission der Medizinischen Universität Wien, permission number: 396/2011, renewed as 063/05/2015) and were in agreement with the Declaration of Helsinki. Informed consent from the next of kin was obtained for the use of brain tissue and for access to medical records.

Brains were collected and processed 2–14 h after death (4.8 h in average, Supplementary table 1, online resource). Both internal carotid and vertebral arteries were cannulated, and the brains were perfused first with physiological saline (1,5 L in 30 min) containing 5 ml of heparin, followed by a fixative solution containing 4% paraformaldehyde, 0.05% glutaraldehyde and 0.2% picric acid in 0.1 M PB, pH 7,4 (4–5 L in 1.5–2 h). During removal and fixation processes, extra care was taken to avoid the deformation of brains. Brainstem blocks (pons + medulla) were removed after perfusion and were postfixed overnight in the same fixative solution but without glutaraldehyde [99]. The blocks were then immersed in phosphate-buffered saline (PBS) (pH 7.4) containing 0.01% sodium azide (Sigma-Aldrich) and 0.02% Bacitracin (Sigma-Aldrich, St. Louis, MO, USA) and were finally stored in this solution long term, pending analysis. Typically, ca. 4 × 4 × 7 mm tissue samples, including the middle part of the LC on one side with surrounding areas, were cut for volume immunostaining. From cases #2, #5, #7, #10, #12 and #14, bilateral samples were processed. Regarding quantifications from the latter samples, average values of the two blocks were calculated.

### Neuropathological analyses of the post-mortem brains

Sampling, diagnostic histological and immunohistochemical stainings as well as neuropathological assessments all followed standard protocols from BrainNet Europe and Brains for Dementia Research UK guidelines [6, 7, 9, 50, 51]. For the first-stage neuropathological assessment of cytoarchitecture and basic cytopathology, 7 μm-thick paraffin sections were cut, dewaxed and stained with hematoxylin/eosin and Luxol fast blue/Nissl. Regarding diagnostic immunohistochemistry, 7 μm-thick paraffin sections were dewaxed and endogenous peroxidase activity was blocked in ethanol containing 1.5% (v/v) H_2_O_2_. Heat-induced epitope retrieval was carried out in microwave oven (5 min. 800 Watt, 2 × 5 min. 250 Watt). After blocking nonspecific antibody binding in 50 mM TRIS-buffered saline (TBS pH 7.4) containing 5% (w/v) low-fat milk powder, the sections were incubated with primary antibodies at room temperature for 70 min. Primary antibodies and their dilutions were the following: mouse monoclonal anti-amyloid-β (Agilent/DAKO,1:100); rat monoclonal anti-phospho-TDP-43 (Ser 409/410; Merck/Millipore, 1:100); rabbit polyoclonal and mouse monoclonal anti-phospho TDP-43 (pSer409/410-1; Cosmo-Bio, 1:1000); mouse monoclonal anti-phospho-tau (Ser 202/Thr205, clone AT8; ThermoFisher/Invitrogen, 1:100); goat polyclonal anti-α-synuclein (R&D Systems, 1:1.000); mouse monoclonal anti α-synuclein (clone 5G4; Roboscreen, 1:2.000); mouse monoclonal anti-p62 (lck ligand; BD Biosciences, 1:200); rabbit polyclonal anti-ubiquitin (DAKO, 1:1.000). Signal detection was performed using the Novolink polymer kit (Leica Biosystems/Novocastra), and nuclear staining was carried out with Mayer’s hematoxylin. For primary goat and rat antibodies, rabbit anti-goat linker IgG (GenWay Biotech) and rabbit anti-rat linker IgG (Vector Laboratories) were used, respectively.

### iDISCO+ tyrosine hydroxylase (TH) and hypophosphorylated tau (AT8) volume co-immunostaining and clearing of human brainstem samples

iDISCO+ volume immunostaining and clearing process were essentially performed as described earlier [3, 78, 79], However, we slightly modified the originally iDISCO+ protocol [78] and applied extended primary/secondary antibody incubations and expanded delipidation time, to compensate the post-mortem effects and high lipid content of the human brainstem tissue, respectively. Briefly, the samples were washed in 0.01 M (1×) PBS 3 times in 5 ml Eppendorf tubes and dehydrated in rising methanol/water series (20–40–60–80–100–100%), 1 h each. Then, the samples were incubated overnight in 66% dichloromethane/33% methanol, washed 2 × 1 h in 100% methanol, for more extensive delipidation. The samples were then bleached with 5% hydrogen peroxide in 100% methanol overnight at + 4 °C, rehydrated in a downgrading series of methanol/water solutions (80–60–40–20–PBS), incubated in permeabilization solution for 4–5 days and then in blocking solution for 2 days, both at 37 °C (0.2% Triton-X100/20% DMSO/0.3 M glycine in 0.01 M PBS + 0.02% sodium azide, 0.2% Triton-X100/10% DMSO/6% normal donkey serum in 0.01 M PBS + 0.02% sodium azide, permeabilization and blocking solutions, respectively). The samples were then incubated with a mixture of primary antibodies for 14 days at 37 °C (anti-TH, rabbit polyclonal, 1:100, Merck-Millipore, #AB152; anti-phospho-tau [Ser202, Thr205], mouse monoclonal, clone AT8, 1:25, Thermo Fisher Scientific, #MN1020) in antibody diluent (0.2% Tween-20/10 μg/ml heparin/5% DMSO/3% normal donkey serum in 0.01 M PBS + 0.02% sodium azide). After extensive washing overnight, the blocks were incubated in a mixture of secondary antibodies (donkey anti-rabbit IgG Alexa Fluor 647, 1:100, Invitrogen, A31573; donkey anti-mouse IgG Alexa Fluor 568, 1:50, Invitrogen, A10037) for another 14 days (diluent: 0.2% Tween-20/10 μg/ml heparin/3% normal donkey serum in 0.01 M PBS + 0.02% sodium azide). Then, the blocks were dehydrated in rising methanol/water series (see above), incubated in 66% dichloromethane/33% methanol for 3 h, in 100% dichloromethane for 2 × 30 min, and were transferred to tubes filled with 100% dibenzyl ether and were stored in this solution.

### Light sheet fluorescence microscopy (LSFM), 3D image reconstruction and quantification processes

A light sheet fluorescence microscope (Ultramicroscope II, Lavision Biotec, Bielefeld, Germany) and the Imspector^™^ 347 software were used. The microscope was equipped with an Olympus MVPLAPO 2x/0.5 objective lens, an Olympus MVX-10 0.63–6.3 × zoom body, a 6.5 mm working distance spherical aberration corrected dipping cap, a sCMOS camera (Andor Neo, pixel size: 2560 × 2160) and Coherent OBIS lasers (488–100 LX, 488 nm; 561–100 LS, 561 nm; 640–100 LX, 640 nm) with appropriate filters. Detailed scanning parameters are reported in the Supplementary Information, online resource.

The serials of 16-bit uncompressed tif images (ca. 3000 Z-levels, ca. 60 GB; raw data; ca. 1500 Z-levels, ca. 30 GB raw data; in case of coronal and horizontal orientation scans, respectively) were then converted to IMS files, using the Imaris File Converter 8.4.2^™^ or 9.2.1^™^ programs (Bitplane, UK), and the 3D vision of acquisitions was reconstructed in the Imaris 9.2.1^™^ or 9.7.2^™^ (Bitplane, UK) software for inspection/quality control, qualitative examination and quantifications.

For the illustrations, snapshot images (1200dpi) were taken from the actual light sheet scans in orthogonal or perspective mode (Imaris). Background subtraction (Imaris) was routinely used for the light sheet scans, and brightness/contrast was slightly adjusted for the snapshots using Photoshop CS6.

3D quantification processes and algorithms applied on LSFM scans are reported in detail in the Supplementary Information, online resource.

### Semiquantitative analysis of AT8^+^ structures

AT8^+^ cellular structures were explored in each coronal LSFM scan with 1.51 μm × 1.51 μm × 2 μm voxel dimensions, applying the highest rendering quality in ‘maximum intensity projection’ (MIP) or ‘blend’ volume rendering, which altogether allowed a reliable discrimination of details on the cellular level. The semiquantitative assessment implied a five-grade score (0 = structure not detected; 0/ +  = only one cell or extremely sparse debris in the entire scan; +  = sparse-, +  +  = moderate-, +  +  +  = high density of examined structure). Summarizing tables in Figs. 4t, 5j and in Supplementary fig. 11 l, online resource, were compiled based on the averages of scores from all examined cases in the respective Braak stages.

### Statistical analyses

*Two-way ANOVA* was used for the evaluation of (i) the AT8^+^ immunosignal volume throughout subregions and Braak stages, (ii) the number of AT8^+^ cell bodies throughout subregions and Braak stages, (iii) the dorso-ventral distribution of AT8^+^ immunosignal volume and number of AT8^+^ cell bodies throughout Braak-stages, and (iv) the AT8^+^ cell body volume vs. process volume throughout Braak-stages in certain subregions. *Paired Student’s t-test* was applied for evaluating the dorso-ventral proportional distribution of AT8^+^ immunosignal volume, number of AT8^+^ and TH^+^ cell bodies as well as the number of dense AT8^+^ cells. *Paired Student’s t test* was also applied for the evaluation of simulated vs. actual nearest neighbour distances in nearest neighbour index (NNI) calculations. *Pearson R correlation test* was used for exploring potential correlations between the age of subjects and the amount of AT8^+^ immunoreactivity/number of AT8^+^ cell bodies. *Analysis of covariance (ANCOVA)* was used to statistically evaluate the impact of age on the accumulation of tau pathology. For the statistical evaluation of all other measurements, including the evaluation of dense and neighbouring cells during in-depth cluster analysis, *one-way ANOVA and Tukey post-hoc tests* were applied. All data were represented and evaluated with acceptable levels of significance set at *P* < 0.05 using the software R - 4.2.1. for ANCOVA and the software *GraphPad Prism 6 for Mac OS X* for all other analyses.

### Movie production

Videos #1–10, online resources, were prepared in mp4 format, using Imaris 9.2.1. ‘video creation’ function with 24 frame/second, 16:9 aspect ratio, 720 kbps bitrates and H.264 codec. All videos have been compressed due to file size restrictions. Original uncompressed videos are available here: https://volume-imaging.com/tau-pathology-of-the-noradrenergic-human-locus-coeruleus-in-3d/high-resolution-versions-of-supplementary-videos-in-the-paper-of-gilvesy-et-al-acta-neuropath-2022/.

## Results

### Study design to investigate tau pathology in the human locus coeruleus - pericoerulear (LC/PC) complex in 3D

We applied the iDISCO + volume immunostaining and clearing technology combined with light sheet fluorescence microscopy (LSFM) to explore cytoarchitecture and tau cytoskeletal pathology of the human LC/PC complex in three dimensions with cellular resolution. Twenty pathologically and neuropathologically extensively characterized subjects were included (six Braak 0, six Braak 1–2, five Braak 3–4 and three Braak 6 brains, all without concomitant α-synuclein and TDP-43-pathology (Supplementary tables 1 and 2, online resources). Fixed tissue blocks with the size of 4 × 4 × 7 mm, containing approximately half of the entire LC (middle part) [73] and adjacent tissue were volume co-immunostained for TH (here, a marker of NA neurons) and hyperphosphorylated tau (AT8).

### 3D delineation and segmentation of the LC/PC complex

**Fig. 1:**
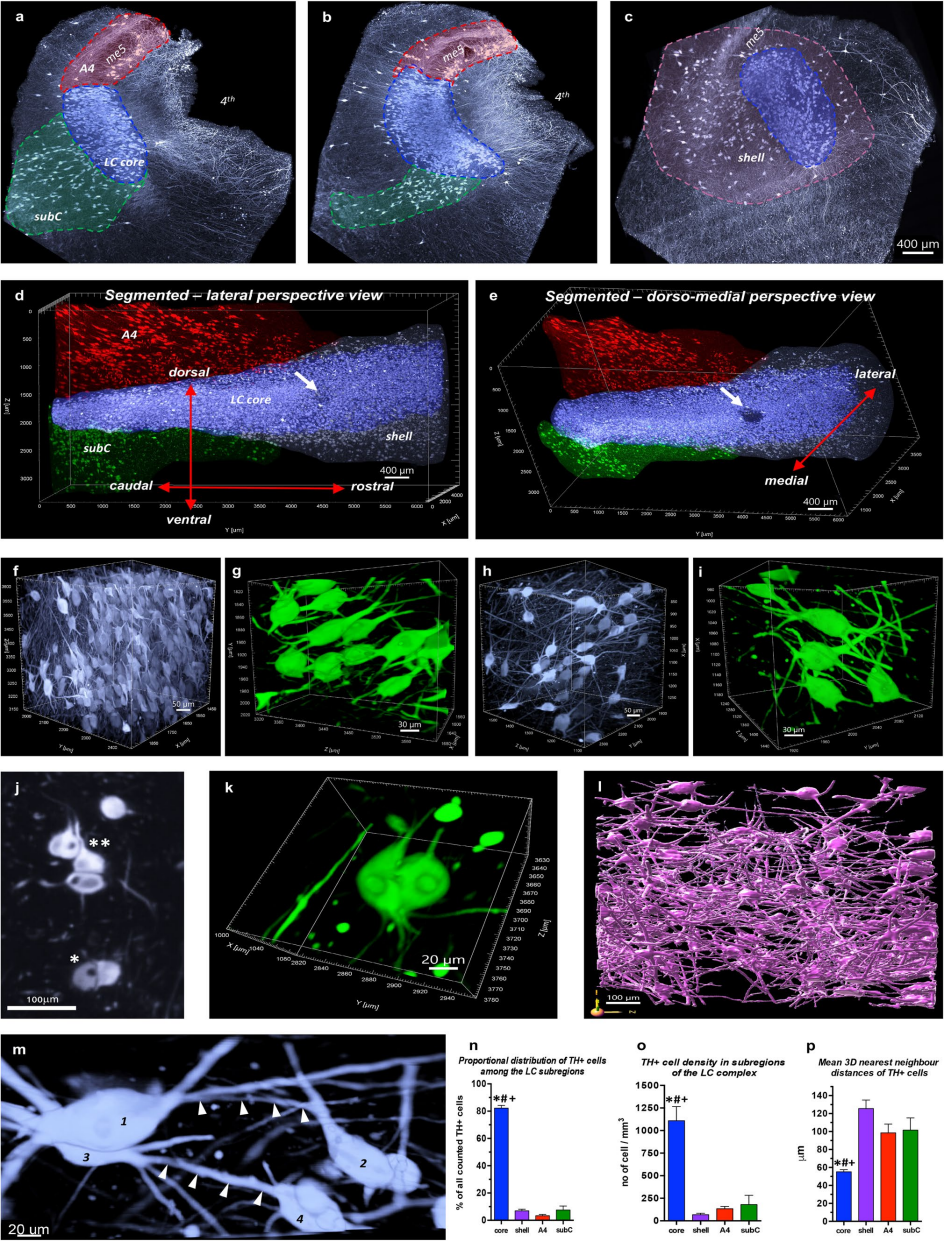
**3D segmentation and cytoarchitecture of the human LC/PC complex.** **a**–**c** 500 μm-thick, representative coronal optical slices with colour-coded segmented subregions. Subcoeruleus (subC): green; locus coeruleus core: blue; A4—pars cerebellaris: red; locus coeruleus shell: pink. me5: mesenchepalic trigeminal tract; 4th: fourth ventricle. **d**–**e** 3D reconstruction of the same block (sagittal view). Arrow: an artery that enters the LC core. **f**–**i** Representative 3D crops (500 × 500 × 500 μm) (**f**, **h**) and higher resolution 3D crops (200 × 200 × 200 μm) in blend rendering mode, detailing the morphology of the somato-dendritic compartment of NA neurons (**g**, **i**) from the caudal (**f**–**g**) and rostral (**h**–**i**) ends of a Braak 0 block. **j** Higher resolution of duo (*) and triplet (**) cells with ‘hugging’ somas from a 20 μm-thick horizontal optical slice. **k** 3D reconstruction of a cell duo with ‘hugging’ somas (blend rendering mode). **l** Reconstruction of the complex dendritic network of LC core NA neurons (surface with 2 μm grain size). **m** Dendro-dendritically closely apposed adjacent TH^+^ neurons (blend 3D rendering mode). Arrows: dendritic appositions between neurons #1– #2 and #3– #4. **n**–**p** Quantifications of TH^+^ cells (Braak 0 brains). *N* = 6; *P < 0.05 (vs. shell), ^#^*P* < 0.05 (vs. A4), ^+^*P* < 0.05 (vs. subcoeruleus). Data expressed as mean ± SEM. Each micrograph shows TH volume immunostaining from Braak 0 cases. Scale bars are indicated in each micrograph

To handle and characterize the large and complex LSFM datasets, first, subregions of the LC/PC complex were 3D segmented based on TH volume staining. NA cells in the human rostral pons are organized as confluent neuronal populations [14], making the reproducible delineation of subregions challenging based on 2D histological methods. At the same time, 3D-reconstructed LSFM scans can be optically sliced in any thickness and in any orientation. Thus, various cell densities, soma sizes, and soma and dendrite orientations were considered during the segmentation of four main subregions of the LC/PC complex in the examined blocks (Fig. 1a–e; Supplementary figs. 1–2, online resources).

LC is a caudo-rostrally elongated tube-like structure with densely packed TH^+^ neurons. In the rostral third of the blocks, the LC became wider and funnel shaped, consisting of a cell-dense ‘*core*’, and a surrounding oval ‘*shell*’ with much lower TH^+^ cell density but with a dense plexus of TH^+^ processes, mainly dendrites (Fig. 1d–e; Supplementary fig. 1, online resource). This ‘pericoerulear dendritic shell’ was described already in early studies [81, 86], and further emphasized in: [75]. In the caudal half of the blocks, the NA neuron population extended latero-ventrally and dorso-medially from the core with a substantially lower cell density. Scattered neurons in the ventro-lateral extension exhibited dorso-ventral soma and dendrite orientations as a main distinguishing feature (vs. the caudo-rostral soma orientation in the LC core), defining the *subcoeruleus* subregion [14, 73]. The dorso-medial extension consisted of sparse TH^+^ cells with distinctly elongated somas around the mesencephalic trigeminal tract (*me5*) and was identified as the *pars cerebellaris of the LC, or A4* [18, 20, 74] (Fig. 1a–e; Supplementary figs. 1–2, online resources).

### Cytoarchitecture of human LC/PC complex subregions

To investigate the spatial distribution of tau pathology with possible implications for the propagation of pathological tau within and from the LC/PC complex, we first studied the cytoarchitecture of this brain region in Braak 0 brains.

Caudal TH^+^ LC core cells are fusiform multipolar neurons with 3–5 dendritic branches and with a predominant rostro-caudal soma orientation (Fig. 1f–g; Supplementary fig. 2a-b and video #1, online resources). Since the LC core cells are densely packed (video #1), it was not possible to trace the complex intermingling dendritic trees and axons of individual neurons based on TH staining. However, AT8 staining of the sparse pretangle-like cells in Braak 0 cases nicely depicted the dendrites, which often bifurcate in the proximity of somas and could even extend 0.8–1.5 mm rostro-caudally and 0.5–1 mm dorso-ventrally and medio-laterally (Supplementary fig. 3, online resource). In many cases, axons could also be traced, sometimes even for millimeters: they were thinner than dendrites and usually less intensely stained (Supplementary fig. 3, online resource). Interestingly, ‘duos’ or ‘triplets’ of close ‘hugging’ TH^+^ somas were frequently noticed in the LC core, as revealed by horizontal optical slicing with a 20 μm step size (Fig. 1j) and by 3D rendering (Fig. 1k).

In the rostral end of the examined blocks, TH^+^ somas in the LC core were wider on average than in the more caudal part of the core, although their length was the same (Supplementary fig. 2a-b, online resource). Also, the rostro-caudal orientation of somas was less obvious at the rostral end of the blocks (Fig. 1h–i). Here, the complex dendritic network was better visualized even based on TH volume staining (Fig. 1l) and, occasionally, dendro-dendritically closely apposed TH^+^ cells were identified (Fig. 1m).

Quantification of TH^+^ cells (Supplementary fig. 4 and ‘TH channel image processing Fiji_ImageJ script’, online resources) revealed that, overall, 82% of all detected NA cells were located in the LC core (Fig. 1n). Segmentation of the tube-shaped LC core to dorsal and ventral halves did not show significant differences in the distribution of TH^+^ cells (Supplementary fig. 5a–c, online resource).

TH^+^ somas in the rostral LC core and in the shell had the same size and morphology, albeit TH^+^ cell density was much lower in the shell (Fig. 1o–p; Supplementary figs. 2a-b and 6a, video #2, online resources). Subcoeruleus TH^+^ neurons intermingled with the long, slender TH^+^ fibers of the dorsal NA bundle and their axons joined this axonal tract (Supplementary fig. 6c–d, online resource). Subcoeruleus TH^+^ neurons exhibited the same shape and size as the caudal LC core cells (Supplementary fig. 2a-b, online resource); however, they showed a dorso-ventral orientation of somas and dendrites and were often organized in small dorso-ventral columns (Supplementary fig. 6e-f, online resource). Somas of A4 TH^+^ neurons were significantly more elongated compared to the LC core cells (Supplementary fig. 2a-b,  online resource), showed rostro-caudal orientation, and their processes formed a complex plexus around the mesenchephalic trigeminal tracts (*me5*) (Supplementary fig. 6 g–i, video #3, online resources)*.* Neighbouring TH^+^ cells with ‘hugging’ somas were frequently noticed in the shell, subcoeruleus and A4 as well (Supplementary fig. 6b, j, online resources).

### Quantification of the AT8^+^ immunostaining volume and AT8^+^ cells in the LC/PC complex throughout the Braak NFT stages

**Fig. 2:**
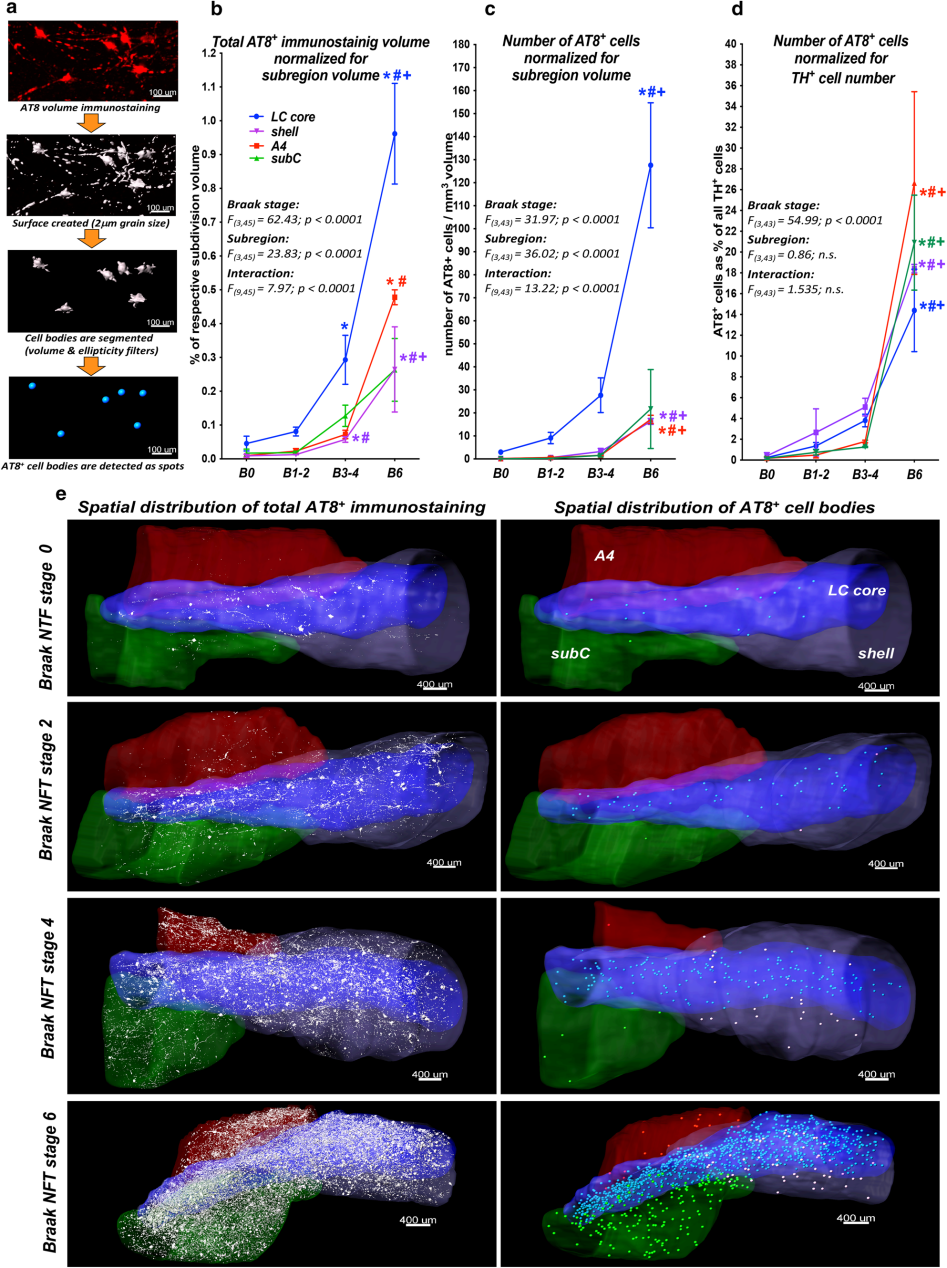
**Large-scale quantitative analysis of the total AT8**^**+**^ **immunostaining volume and AT8**^**+**^ **cells**. **a** Analysis pipeline. **b**–**d** Summary and graphical representation of quantified AT8^+^ immunostaining. AT8^+^ immunostaining volumes normalized for subregion volumes (**b**). Number of AT8^+^ neurons normalized for subregion volume (**c**) and for TH^+^ cell number (**d**). Two-way ANOVA main effect *F*, *dF* and *P* values are indicated in each panel. *N* = 6 (Braak 0), *N* = 6 (Braak 1–2), *N* = 5 (Braak 3–4), *N* = 3 (Braak 6); *P < 0.05 (vs. Braak 0), ^#^*P* < 0.05 (vs. Braak 1–2), ^+^*P* < 0.05 (vs. Braak 3–4). Data expressed as mean ± SEM. **e** Representative images demonstrating full tau staining volumes (white in the left column) and AT8^+^ cells (as dots in the right column). Subregion volumes are colour-coded as shown in the upper right panel. subC: subcoeruleus. B0, B1-2, B3-4, B6 = Braak NFT stages 0, 1–2, 3–4, 6, respectively. Scale bars are indicated in each micrograph

We started the characterization of LC tau cytoskeletal pathology by quantifying the total AT8^+^ immunosignal volume and the number of AT8^+^ cell bodies (Fig. 2a). The total AT8^+^ immunostaining volume, adjusted for subregion volume, increased with the Braak stages. It doubled between Braak 0 and 1–2, tripled between Braak 1–2 and 3–4, and tripled again between Braak 3–4 and 6 in the LC core (Fig. 2b, e). It gradually increased with the Braak stages also in the shell, A4 and subcoeruleus; however, to a significantly lower degree, compared to the LC core (approximately 1/3 of the core AT8^+^ signal), throughout the Braak stages (Fig. 2b; Supplementary fig. 7a, online resource).

The number of AT8^+^ cell bodies, adjusted for subregion volume, revealed a highly similar trend, but increased dramatically, almost 5 × between Braak 3–4 and 6 in the LC core (Fig. 2c, e). The number of AT8^+^ cells in the PC subregions was low until Braak 3–4, increased remarkably only in Braak 6, but remained much lower (1/8 – 1/16) compared to the LC core throughout the Braak stages (Figs. 2c; Supplementary fig. 7b, online resource). However, when the number of AT8^+^ cells was normalized to the TH^+^ cell number, the gradual increase of AT8^+^ cell number with the Braak stages was still obvious, but no differences between the subregions were noted (Fig. 2d; Supplementary fig. 7c, online resource). These findings indicate that the significantly higher total number of AT8^+^ cells in the LC core, compared to the PC regions, is the consequence of the much higher NA cell density in the LC core.

The potential impact of the subject’s age on the accumulation of tau pathology was explored with covariance analysis in the LC core. Despite the age of subjects at death increased with the Braak stages (Supplementary fig. 8a, online resource), ‘age’ covariate per se did not significantly influence the full volume of AT8^+^ immunostaining or the number of AT8^+^ cell bodies, in sharp contrast to the Braak stage (Supplementary fig. 8b, online resource). Moreover, there was no significant correlation between the age of subjects at death and any quantified parameters of AT8^+^ immunoreactivity (Supplementary fig. 8c–h, online resource).

### Spatial relation of AT8^+^ cell bodies and processes

**Fig. 3:**
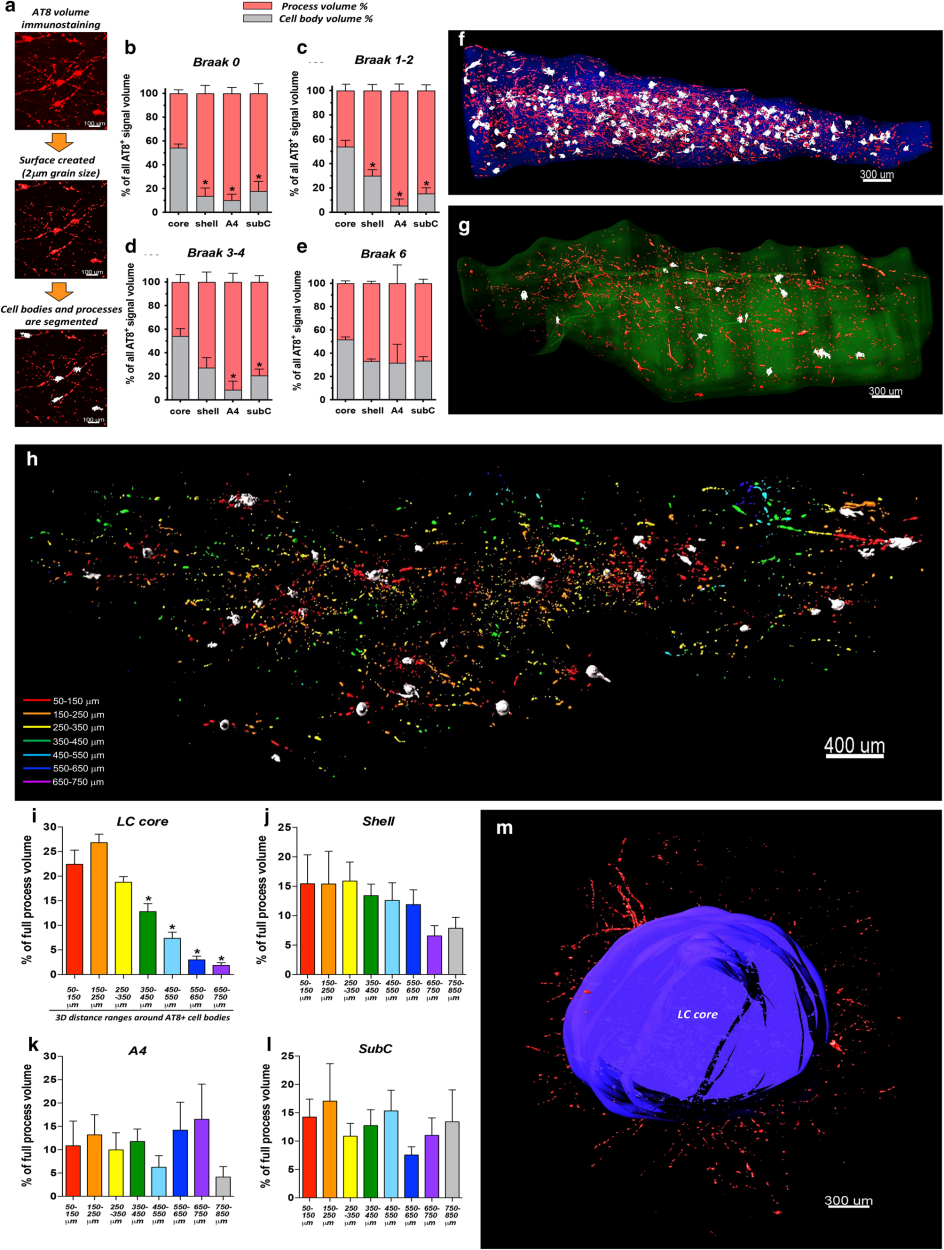
**Spatial relation of AT8**^**+**^ **cell bodies and processes**. **a** Segmentation pipeline of AT8^+^ cell bodies and processes. **b**–**e** Proportional distribution of AT8^+^ volumes for cell bodies and processes in LC/PC subregions throughout the Braak stages. *N* = 6 (Braak 0), *N* = 6 (Braak 1–2), *N* = 5 (Braak 3–4), *N* = 3 (Braak 6). *P < 0.05 (vs. LC core cell body volume %). Data expressed as mean ± SEM. **f**–**g** Representative images demonstrating the segmented AT8^+^ cell body volumes (white surface) and process volumes (red surface) in the LC core (transparent blue surface in **f**) and in the subcoeruleus (transparent green surface in **g**) from a Braak 3 subject. **h** Representative image demonstrates in-depth spatial analysis of processes around cell bodies in the LC core in a Braak 0 subject. The 3D space around cell bodies (white surfaces) was divided into concentric, 100 μm-wide spheres, and the AT8^+^ process volume was determined in each sphere. The portions of process volumes in certain spheres are colour-coded as indicated in the lower left corner. **i**–**l** Quantification of the spatial distribution of AT8^+^ processes around AT8^+^ cell bodies in the LC core (**i**), shell (**j**), A4 (**k**) and subcoeruleus (**l**) in Braak 0–2 subjects. *N* = 12; *P < 0.05 (vs. the 50–150 μm-range values). Data expressed as mean ± SEM. **m** Illustration of AT8^+^ processes (red) that leave the LC core (blue surface); coronal perspective from caudal view in a Braak 2 subject. B0, B1-2, B3-4, B6 = Braak NFT stages 0, 1–2, 3–4, 6, respectively. Scale bars are indicated in each micrograph

To gain a more complete overview of the spatial distribution and origin of AT8^+^ processes in the various LC/PC subregions throughout the progression of AD, as the next step, we segmented the volumes of AT8^+^ cell bodies and the volumes of neurites (Fig. 3a) and systematically compared their proportions and spatial relations. The AT8^+^ cell body volume/process volume ratio was ca. 1:1 in the LC core and it did not change with the more advanced Braak stages (Fig. 3b–f). In sharp contrast, in the shell, A4 and subcoeruleus, process volumes dominated over the cell body volumes, providing 70–95% of the total AT8^+^ immunosignal volume, while the proportion of cell body volume was only 5–30%. This applied for all Braak stages, albeit the proportion of cell body volume showed a slightly increasing tendency with more advanced stages (Fig. 3b–e, g). We also studied the spatial distribution of AT8^+^ processes around the cell bodies in early (0–2) Braak stages (this analysis was not possible in more advanced stages, due to the high density of AT8^+^ structures). Namely, the 3D space was divided into 100 μm-thick concentric sphere-like zones around the cell bodies and the volume of AT8^+^ processes was determined in each sphere (Fig. 3h). In the LC core, process volumes showed a gradual concentric decrease from the spots representing the center of AT8^+^ cell bodies (Fig. 3i). In contrast, this ‘zoning’ was not noticed in the PC subregions, indicating that many AT8^+^ processes could not be traced back to a local cell body. (Fig. 3j–l). All these quantitative data, together with the three-dimensional visual observation, indicate that the vast majority of AT8^+^ processes in the LC core originate from local neurons. In contrast, the majority of AT8^+^ processes in the shell, A4 and subcoeruleus arise from neurons in the LC core (Fig. 3m).

### Heterogeneity and semiquantitative assessment of AT8^+^ cellular structures throughout the Braak-stages

**Fig. 4:**
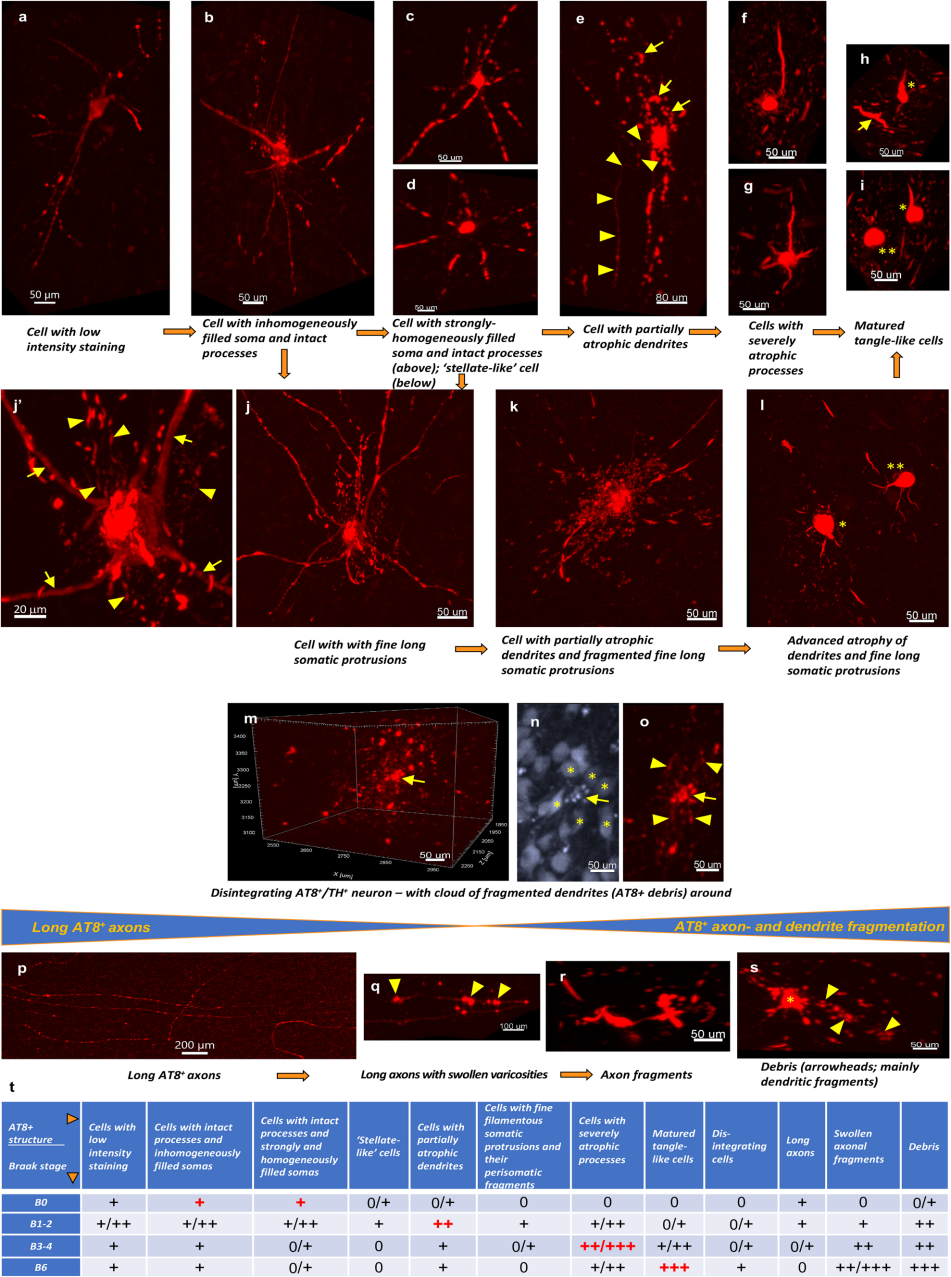
**AT8**^**+**^ **cellular structures in 3D and their semiquantitative assessment.** **a**–**s** Representative images of AT8^+^ structures on the cellular level, identified by volume imaging. The orange arrows indicate a hypothetical direction of neurofibrillary tangle maturation. Cell with low-intensity staining (Braak 2 subject) (**a**). Cell with intact processes and inhomogeneously filled soma (Braak 0 subject) (**b**). Cell with intact processes strongly and homogeneously filled soma (Braak 2 subject) (**c**). ‘Stellate-like’ cell (Braak 2 subject) (**d**). Cell with partially atrophic dendrites (Braak 2 subject); arrows: atrophic, swollen dendritic branch; arrowheads: morphologically intact axon (**e**). Cells with severely atrophic processes (Braak 3 subjects) (**f**–**g**). Matured tangle-like, flame-shaped cell (*); arrow: swollen axon fragment; (Braak 6 subject) (**h**). Matured tangle-like cell with flame-like morphology (*), matured tangle-like cells with ball-like morphology (**), (Braak 6 subject) (**i**). Cell with fine filamentous somatic protrusions (Braak 2 subject) (**j**). Same cell as in j but shown in 2D projection and higher resolution; arrows: dendrites; arrowheads: fine somatic protrusions (**j’**). Transition form with fragmenting somatic protrusions (Braak 2 subject) (**k**). One cell with advanced fragmentation of processes and somatic protrusions (*) and another cell with advanced process atrophy (**), (Braak 3 subject) (**l**). Disintegrating cell/soma (arrow), (Braak 2 subject) (**m**). Same cell as in m but shown in 2D projection with higher resolution (TH channel, gray); arrow: disintegrating soma; *adjacent intact TH^+^ cells (**n**). Same cell as in m and n but shown in 2D projection with higher resolution (AT8 channel, red); arrow: disintegrating soma; arrowheads: fragmenting dendrites (**o**). Long, morphologically intact axons (Braak 0 subject) (**p**). Long axon with swollen varicosities (Braak 0 subject); arrowheads: swollen varicosities (**q**). Swollen axonal fragments (Braak 3 subject) (**r**). Debris of fragmenting dendrites from a Braak 2 subject; *cell body of AT8^+^ cell; arrowheads: fragmenting dendrites of the same cell (**s**). (**t**) Semiquantitative scoring of the cellular AT8^+^ structures. This table is the summary of Supplementary table 3. The dominating cellular forms in certain Braak stages are indicated with red marks. Scale bars are indicated in each micrograph

AT8^+^ structures were studied in 3D at the cellular level in more detail with the application of higher resolution coronal orientation LSFM scans, first, in the LC core (Supplementary fig. 9, videos #4–7, online resources). The following categories were determined, which were characterized by both qualitative (Fig. 4) and quantitative (Supplementary fig. 10, online resource) evaluation: (i) ‘cells with low-intensity staining’ with homogenous and faint AT8^+^ volume staining of cell bodies as well as morphologically intact dendrites and axons (Figs. 4a). (ii–iii) ‘Cells with inhomogeneously filled soma and intact processes’ and ‘cells with homogeneously filled soma and intact processes’, where the cell bodies were inhomogeneously or homogeneously filled by strong AT8^+^ volume staining, which, in addition, nicely depicted the morphologically intact dendrite arborisations and axons (Fig. 4b–c). The shape, size and distribution of intensively stained AT8^+^ granules in the inhomogeneously filled somas were highly variable, possibly reflecting on the aggregation status of hyperphosphorylated tau in these cell bodies (Supplementary fig. 11a–e, online resource). (iv) Cells with homogeneously filled somas rarely exhibited ‘stellate-like’ morphology in 3D, where the AT8^+^ dendrites were only faintly stained close to the cell body, whereas the staining was strong in more distal dendritic segments (Fig. 4d; Supplementary fig. 13a, online resource). These cells had enlarged somas with particularly strong immunostaining intensity, compared to other cellular categories (Supplementary fig. 10b–c, online resource). (v) ‘Cells with partially atrophic dendrites’ with strongly and homogeneously filled cell bodies and seemingly intact axons, but with a swollen, partially atrophic dendritic tree. The atrophy was restricted to a certain dendritic branch only or to distal parts of more branches (Fig. 4e; Supplementary fig. 12d, online resource). (vi) ‘Cells with severely atrophic processes’ with strongly filled soma but with heavily trimmed-truncated dendrite branches and without detectable axons (Fig. 4f–g). (vii) ‘Mature tangle-like cells’ with ball- or ‘flame’-like morphology but without remaining processes in any particular 3D direction (Fig. 4h–i). According to quantitative measurements, in agreement with the 3D visual inspection, full cell volume, process volume, full cell surface area as well as the number of dendritic branches were gradually decreased in the order of ‘cells with partially atrophic dendrites’ — ‘cells with severely atrophic processes’ — ‘mature tangle-like cells’, compared to other cellular categories (Supplementary fig. 10b-c, online resource). (viii) A small subset of cells with inhomogeneously or homogeneously labeled somas displayed dense, 1–2 μm thin, long filamentous protrusions (in contrast to the 3–4 μm thick proximal dendrites), which typically extended concentrically 50–250 μm around the cell bodies but rarely even longer (Fig. 4j–j’). These somatic protrusions often showed a swollen, fragmented morphology with the 3D appearance of a perisomatic ‘cloud’ of debris (Fig. 4k). In more advanced cellular degeneration, it was not possible to discriminate between the degenerating proximal dendrite branches and the swollen-fragmenting somatic protrusions (Fig. 4l). Due to the dense somatic protrusions, the full cell surface area of these cells was especially extensive, compared to other cellular forms (Supplementary fig. 10b–c, online resource). (xi) ‘Disintegrating cells’ with fragmenting cell bodies surrounded by heavily fragmented and swollen dendrite debris, where the shape of the individual dendrites was still recognizable in 3D. The soma of these neurons showed fragmenting morphology also in the TH channel (Fig. 4m–o).

In addition, sparsely, several presumptive transition forms and variations of the cell pathological themes listed above were noticed, like transitions between cells with low-intensity staining and inhomogeneously filled cells (Supplementary fig. 11f, online resource); partially fragmented somatic protrusions (Supplementary fig. 11g–h, online resource); cells with inhomogeneously filled somas and severely atrophic processes (Supplementary fig. 11i, online resource); cells with almost matured tangle-like morphology but with still visible fragmented somatic protrusions (Supplementary fig. 11j, online resource); or cells only exhibiting short proximal chunk of degenerating dendrites, close to tangle-like cells in morphology (Supplementary fig. 11k, online resource). Taken together, 3D analysis reveals an intriguingly high morphological complexity of cellular tau pathology in the LC core, which is rather difficult to comprehensively explore in 5–7 μm optical slices corresponding to the traditional histological section thickness, due to the limited possibility for tracing back processes to their cell bodies (Supplementary figs. 12–13, online resources).

Semiquantitative assessment of various AT8^+^ cellular forms in the LC core throughout the Braak stages demonstrated that the dominant forms were cells with homogeneously or inhomogeneously filled somas and intact processes in Braak 0, cells with partially atrophic dendrites in Braak 1–2, cells with severely atrophic processes in Braak 3–4 and mature tangle-like cells in Braak 6 (Fig. 4t; Supplementary table 3, videos #4–7, online resources). However, cells with partial dendritic atrophy were rarely observed already in four out of six Braak 0 LC samples. Moreover, the presumably earliest AT8^+^ cellular forms (like cells with low-intensity staining, cells with inhomogeneously filled soma and intact processes) were present in all stages until Braak 6, suggesting a progressive hyperphosphorylated tau formation in the LC up till the most advanced stage of AD. Pretangle-like cells with inhomogeneously filled soma and morphologically intact processes were identified in all six Braak 0 samples. Moreover, visual inspection of 3D-rendered cells linked virtually all AT8^+^ processes (axons or dendrites) to a corresponding AT8^+^ cell body in all Braak 0 cases. Cells with fine filamentous somatic protrusions and/or their perisomatic fragments were sparsely noticed in Braak 1–2, very rarely in Braak 3–4, but were virtually absent in Braak 0 or 6 (Fig. 4t; Supplementary table 3, online resource).

For the more comprehensive 3D characterization of tau cytoskeletal pathology, we defined and scored three more categories of AT8^+^ structures in the semiquantitative assessment, focusing on processes: (i) ‘long, AT8^+^ axons’, which were either morphologically intact or exhibited swollen axonal varicosities suggesting impaired axoplasmic transport [2, 4, 5] (Fig. 4p–q). These axons could be traced back to their cell bodies (Supplementary fig. 3, online resource). (ii) ‘Swollen axon fragments’ (Fig. 4r), and (iii) pieces of ‘debris’, which, according to the 3D visual observation, may have resulted primarily from dendritic fragmentation (Fig. 4s). Long axons were less and less abundant, while the amount of axonal fragments and debris increased with the more advanced Braak stages (Fig. 4t; Supplementary table 3, online resource).

In the PC regions (shell, A4, subcoeruleus), the same AT8^+^ cellular forms were detected like in the core, however, with less abundance. This was in agreement with the much lower density of TH^+^ neurons (and hence AT8^+^ cells) in these regions in general (Supplementary fig. 11 1, online resource) and suggests that NA neurons in the PC regions exhibit the same cellular tau cytoskeletal pathology as those in the core with higher cell density.

### 3D analysis reveals more advanced tau pathology in the dorsal vs. ventral segment of the LC core

**Fig. 5:**
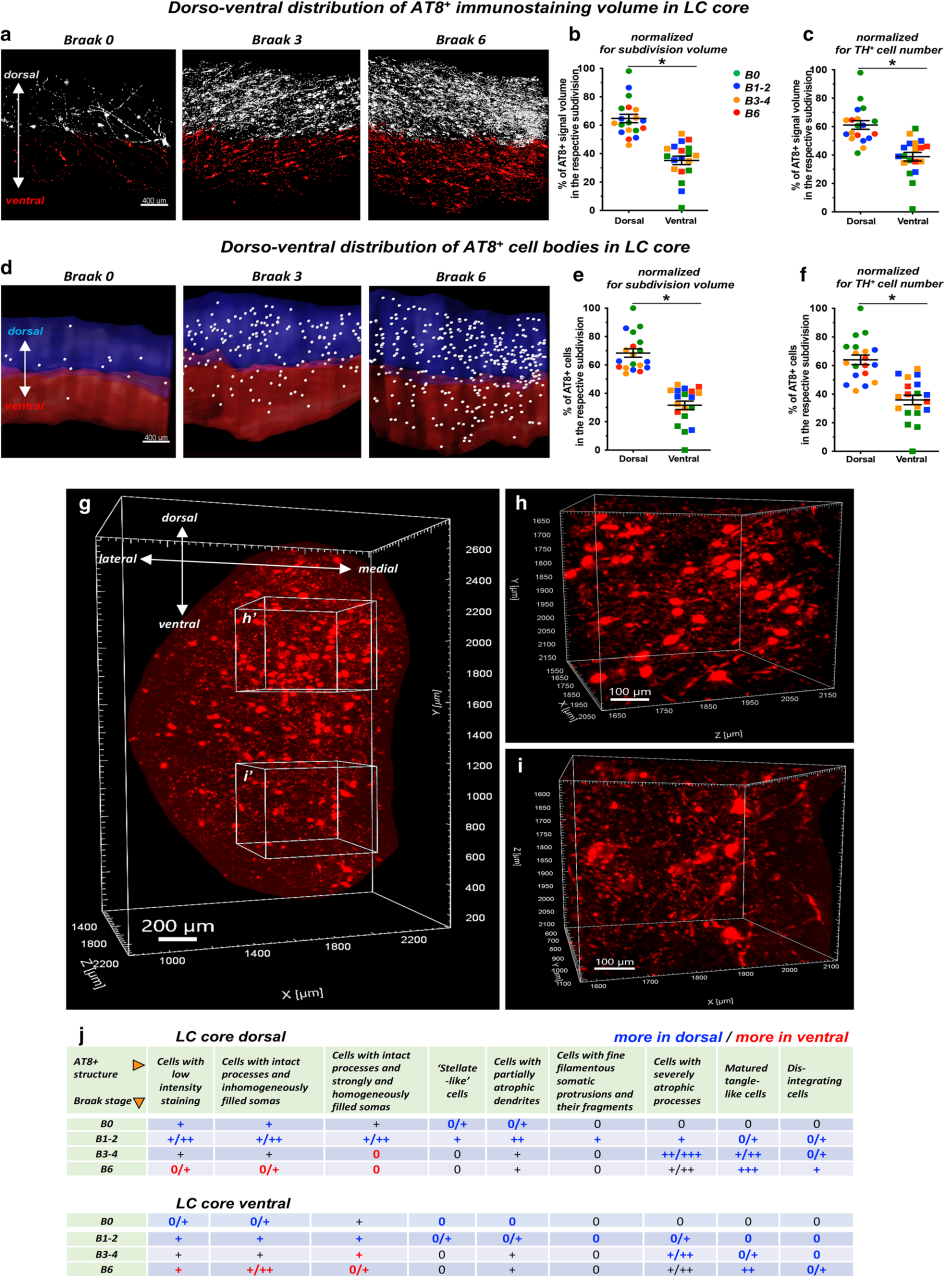
**More advanced tau pathology in the dorsal vs. ventral part of the LC core.** **a** AT8^+^ immunostaining is more abundant in the dorsal part of the LC core (white surface) than in the ventral part (red surface) throughout the Braak stages. **b**–**c** Proportional distribution of AT8^+^ immunostaining volume along the dorso-ventral axis, normalized for segment volume (**b)** and TH^+^ cell number (**c**). *N* = 19; *P < 0.05. Data are represented as individual data points and as mean ± SEM. **d** More AT8^+^ cells (white dots) are found in the dorsal LC core segment (blue surface), compared to the ventral LC core segment (red surface). **e**–**f** Proportional distribution of AT8^+^ cells along the dorso-ventral axis, normalized for segment volume (**e**) and TH^+^ cell number (**f**). *N* = 19; *P < 0.05. Data represented as individual data points and as mean ± SEM. **g**–**i** Demonstration of the dorso-ventral distribution of AT8 volume staining in a 1000 μm-thick optical slice of LC core (Braak 6). Boxes in **g** indicated with **h’** and **i’** are enlarged in **h** and **i**, respectively. (**j**) Semiquantitative scoring of cellular AT8^+^ structures in the dorsal (upper table) and ventral (lower table) LC core segments. Blue marks: structure is more abundant in the dorsal segment. Red marks: structure is more abundant in the ventral segment. B0, B1-2, B3-4, B6 = Braak NFT stages 0, 1–2, 3–4, 6, respectively. Scale bars are indicated in each micrograph

To explore the spatial distribution of hyperphosphorylated tau, we focused on the LC core containing 82% of all detected NA cells. First, the tube-like LC core was further segmented into equal-sized, dorsal–ventral divisions, and the distribution of AT8^+^ structures was quantified in both segments. After normalizing the data both to segment volumes and TH^+^ cell counts, both total AT8^+^ immunostaining volume and the number of AT8^+^ cell bodies were proportionally higher in the dorsal, compared to the ventral segment, irrespectively of the Braak-stage (Fig. 5a–f; video #8, online resource; detailed two-way ANOVA values are reported in Supplementary table 4, online resource). Moreover, semiquantitative scoring of AT8^+^ structures separately in the dorsal and ventral segments revealed that cells with low-intensity staining, cells with inhomogeneously or homogeneously filled soma and intact processes, and cells with partially atrophic dendrites were more abundant in the dorsal, compared to the ventral segment in the earlier Braak stages 0–2. However, in late stage 6, these presumably early AT8^+^ cellular forms were more frequent in the ventral vs. the dorsal segment. In contrast, mature tangle-like cells in the more advanced Braak stages 3–6 were more abundant in the dorsal, compared to the ventral segment (Fig. 5g–j). These findings suggest that tau pathology is more advanced in the dorsal vs. the ventral portion of the human LC core throughout the Braak stages, not only in quantity of AT8^+^ staining volume- and cell body numbers but also in terms of the composition of AT8^+^ cellular structures.

### Spatial distribution of AT8^+^ cells in the LC core exhibits a clustering tendency

**Fig. 6:**
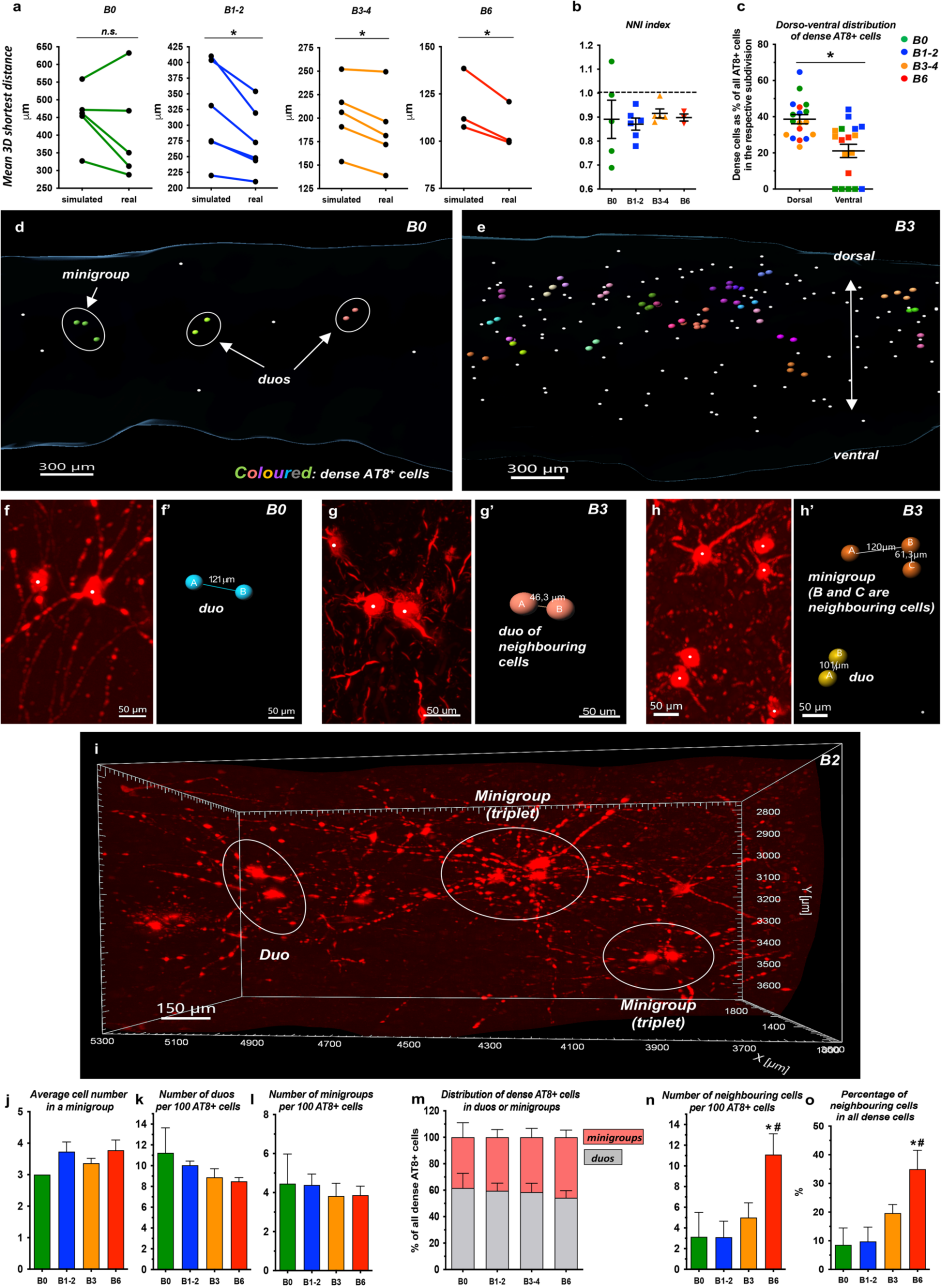
**In-depth spatial analysis reveals a clustering tendency of AT8**^**+**^ **cells in the LC core. a** Paired comparisons of Monte-Carlo simulated and actual (real) mean nearest neighbour distances throughout the Braak stages. *N*= 5 (Braak 0), *N*  = 6 (Braak 1–2), *N* = 5 (Braak 3–4), *N* = 3 (Braak 6); *P < 0.05. **b** Graphical representation of nearest neighbour index (NNI) calculations. Cases with values below 1.0 exhibit a clustering tendency of AT8^+^ cells. Data are represented as individual data points and as mean ± SEM. **c** Distribution of dense AT8^+^ cells along the dorso-ventral axis; *N* = 18; *P < 0.05. Data are represented as individual data points and as mean ± SEM. **d**–**e** Demonstration of dense cells (big coloured spots) and non-dense cells (small white dots) in the volume of a Braak 0 (**d**) and a Braak 3 (**e**) case. Dense cells were clustered and colour-coded by the ‘split spot’ MATLAB Imaris XTension: cells that belong to the same ‘duo’ or ‘minigroup’ have the same colour. **f**–**h** Examples of dense (clustering) cells. AT8 channel, white dots indicate cell bodies (**f**, **g**, **h**). Coloured spots in **f’**, **g’** and **h’** refer to the AT8^+^ cells in panels **f**, **g** and **h**, respectively. Distances between the centers of cell bodies (in μm) are indicated in **f’**, **g’** and **h’**. **i** Volume rendering of AT8^+^ neurons with an indication of dense cells, representative 3D crop (1.8 × 1 × 1 mm overview, Braak 1 brain). **j**–**o** Quantitative characterization of dense cells. *N* = 5 (Braak 0), *N* = 6 (Braak 1–2), *N* = 5 (Braak 3–4), *N* = 3 (Braak 6); *P < 0.05 (vs. Braak 0); ^#^*P* < 0.05 (vs. Braak 1−2). Data expressed as mean ± SEM. Scale bars are indicated in each micrograph

Since visual inspection indicated multiple seemingly clustered AT8^+^ cells in the LC core (Supplementary fig. 9b–c, online resource), we further explored the spatial distribution of AT8^+^ cells applying in-depth 3D cluster analysis. The observed (real) mean 3D nearest neighbour distance of AT8^+^ cells in the LC core was indeed significantly shorter than the average distance between nearest neighbours in a Monte-Carlo simulated hypothetical random distribution of cells in the same volume in Braak stages 1–2, 3–4 and 6 but not in stage 0 (Fig. 6a). Nearest neighbour indexes (NNI) were calculated to 0.89 ± 0.07, 0.87 ± 0.02, 0.91 ± 0.01 and 0.89 ± 0.01 in Braak 0, Braak 1–2, Braak 3–4 and Braak 6 groups respectively, indicating a clustering tendency of AT8^+^ cell bodies in all Braak stage groups (Fig. 6b; ‘NNI calculation MATLAB script’, online resource).

To analyze the clustering tendency at the individual cell level, we defined ‘*dense (clustered) AT8*^+^ *cells’* that were spatially closer to each other than 75% of the average 3D nearest neighbour distance. Significantly more dense cells were noted in the dorsal vs. the ventral segment of the LC core (Fig. 6c). As the next step, the spatial distribution of dense cells was further characterized by the ‘split spot’ MATLAB XTension (Supplementary fig. 14, online resource). This analysis revealed that dense cells were present either as part of ‘*duos*’ or ‘*minigroups*’ (i.e. 3–9 clustering cells) (Fig. 6d–j; video #9, online resource). Interestingly, mini groups including more than 4 cells often had the shape of a string of pearls, instead of a compact knot (Supplementary fig. 15a–a’, online resource). In addition, we determined the subset of those dense cells that were in equal or shorter distance to the nearest neighbouring AT8^+^ cell than the average nearest neighbour distance of TH^+^ cells in the actual scan. Since virtually all AT8^+^ cells were also TH^+^ in the LC core, this subset defined the (statistically) *immediate neighbouring AT8*^+^ *NA neurons (‘neighbouring AT8*^+^ *cells’)* (Figs. 6g–g’, h–h’). The number of duos and mini groups calculated to 100 AT8^+^ cells, as well as the proportion of duo- or mini group-forming dense cells were stable throughout the Braak stages (Fig. 6k–m). In contrast, the number of ‘neighbouring AT8^+^ cells’, also if calculated to 100 AT8^+^ cells, showed an increasing tendency in Braak 3–4 and a significant increase in Braak 6 (Fig. 6n–o).

Duos or mini groups were composed of any types of AT8^+^ cellular forms from ‘cells with low-intensity staining’ to ‘mature tangle-like cells’, depending on the actual Braak stage, and no particular cellular form was enriched among dense cells (Supplementary fig. 16a-e, video #9, online resources).

### 3D imaging reveals dendro-dendritically apposed AT8^+^ cells

**Fig. 7:**
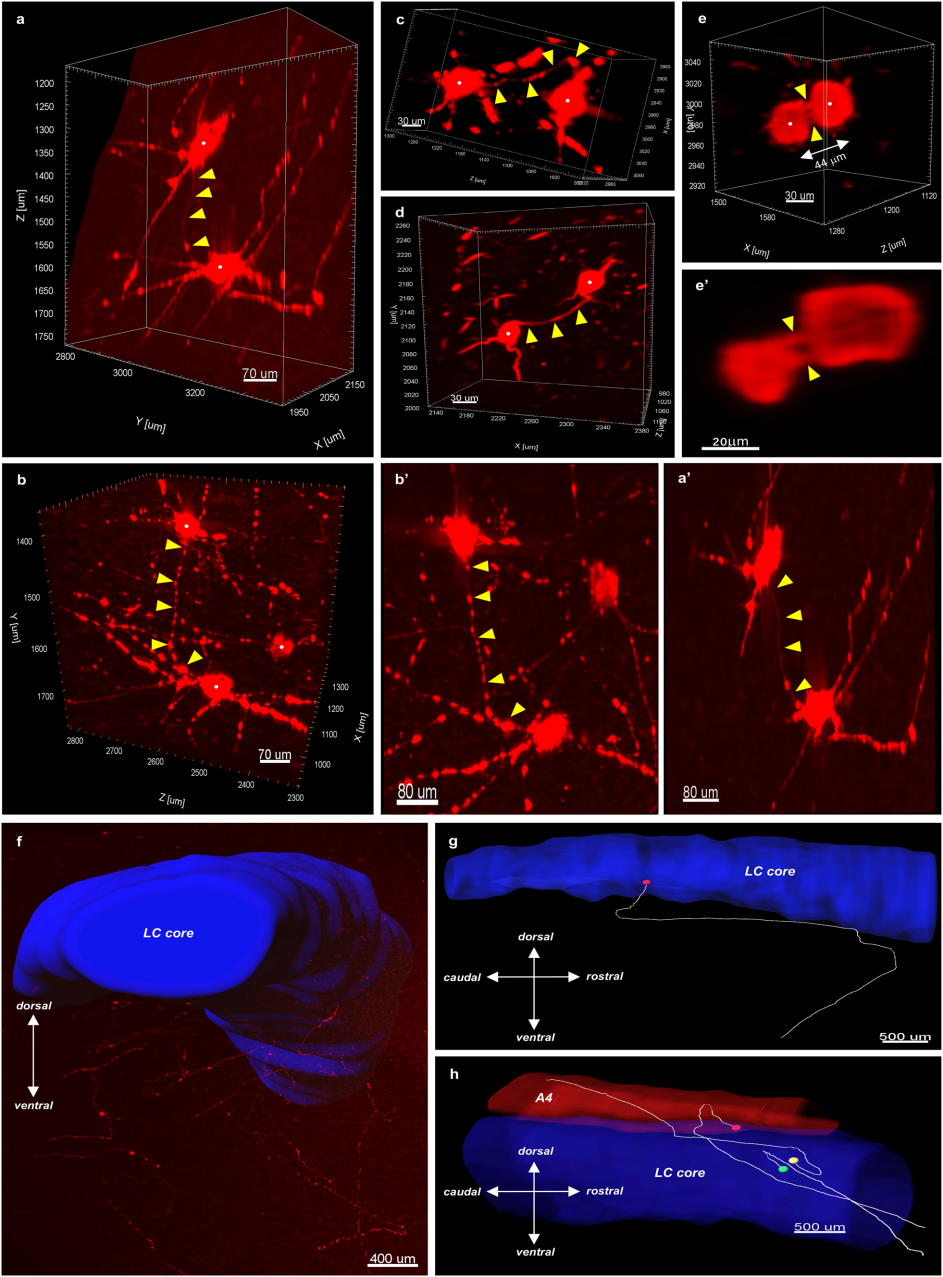
**Potential routes for spreading of hyperphosphorylated tau: AT8**^**+**^ **cell appositions and long axons. a**–**d** Dendrites from pairs of AT8^+^ neurons that are closely apposed in the LC core from two Braak 0 subjects (**a**–**a’** and **c**), from a Braak 1 subject (**b**–**b’**) and from a Braak 6 subject (**d**). **a’** and **b’** are 2D-projected representations of the 3D-rendered cells shown in **a** and **b**, respectively. Arrowheads in all panels point to the dendritic appositions. (**e**–**e’**) 3D rendering of two neighbouring duo cells with apparent ‘tunnel-like’, thin interconnections (arrowheads) (**e**) and a 2 μm-thick optical slice showing the same cells (**e’**). (**f**) AT8^+^ axons arising from the LC core (blue surface) and joining the dorsal NA bundle ventrally from the LC. **g**–**h** Traced AT8^+^ axons of LC core neurons. Axons from cell bodies (coloured dots) usually take a U-turn before leaving the LC core, typically in the ventral direction, running towards the dorsal NA bundle (‘red’ cell in g, and ‘red’ and ‘green’ cells in h), or rarely dorsally, towards the A4 (‘yellow’ cell in h). Scale bars are indicated in each micrograph

Closer 3D visual inspection of dense cells revealed occasional dendro-dendritically closely apposed neurons, where dendritic branches from two adjacent AT8^+^ cells came in close contact. These were typically ‘duo’ cells with inhomogeneously or homogeneously filled somas and intact processes, especially in the Braak 0–2 cases (Fig. 7a–a’, 7b-b’, 7c). However, sparse AT8^+^ cells with partially atrophic dendrites or with heavily atrophic processes were in close dendritic apposition as well, even in Braak 6 cases (Fig. 7d). Moreover, AT8^+^ ‘neighbouring’-type duo cells rarely exhibited short thin ‘tunnel-like’ interconnections (Fig. 7e–e’).

### AT8^+^ long axons leave the LC and join to the dorsal NA bundle already in Braak stage 0

In the early Braak 0–2 stages, AT8^+^ axons could often be identified ventrally from the LC core (Fig. 7f). These axons originate from AT8^+^ LC core cells with inhomogeneously or homogeneously filled soma. These long axons could be traced individually from the cell bodies even for 3–6 mm long, until the edges of the tissue blocks. They typically made a U-turn at a few hundred μm distance from their cell bodies, left the LC core and joined the central tegmental tract (dorsal NA bundle) running ventrally and ventrolaterally from the LC core in the caudo-rostral direction (Fig. 7g–h). AT8^+^ cells in the dorsal LC core rarely sent axons dorsally, in this case towards the A4 (Fig. 7h, ‘yellow’ cell body).

### Dendrites of AT8^+^ LC NA cells extend into the subependymal NA plexus already in Braak stage 0

**Fig. 8:**
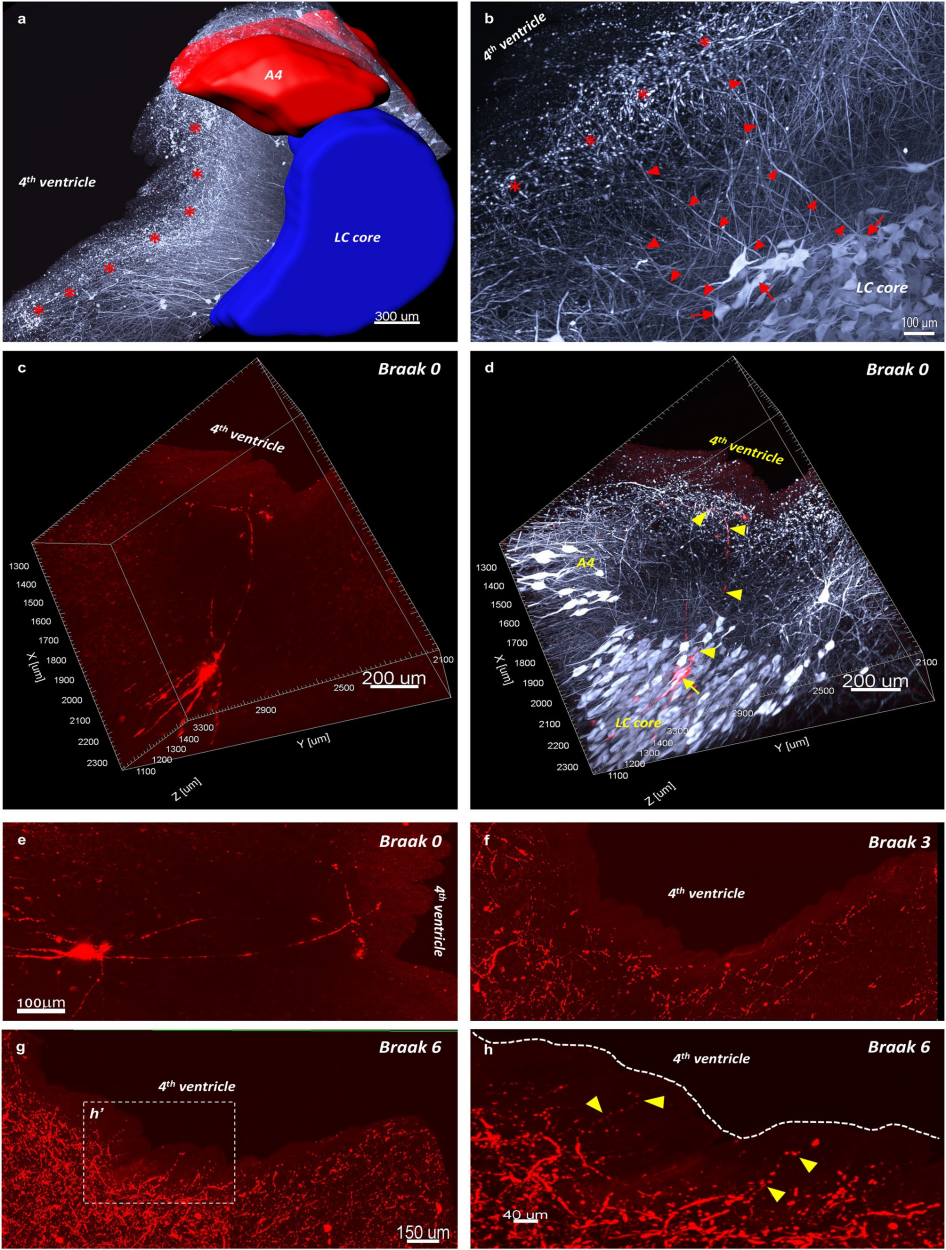
**AT8**^**+**^ **LC dendrites join the subependymal NA plexus below the 4th ventricle. a**–**b** The subependymal NA plexus below the 4th ventricle with terminal dendritic segments (*) and dendrites (arrowheads in **b**) from LC NA cell bodies (arrows in **b**), TH channel. **c**–**d** AT8^+^ NA neuronal soma (arrow in **d**) in the LC core extending hyperphosphorylated tau-filled dendrite (arrowheads in **d**) to the subependymal NA dendritic plexus (Braak 0 subject). Red and whitish grey represent AT8 and TH volume staining, respectively. **c**: AT8 channel only;** d**: AT8 + TH channels together. **e** 2D projection of the AT8^+^ neuron shown in **c**, AT8 channel. **f**–**h** Dense hyperphosphorylated tau-filled subependymal dendrites in 300 μm-thick optical slices, in a Braak 3 (**f**) and a Braak 6 (**g**–**h)** subject. Box indicated with **h’** in **g** is enlarged as **h**. Hyperphosphorylated tau-filled terminal dendritic segments (arrowheads in **h**) approach the ventricle surface to 40–50 μm. The dashed line in **h** indicates the ventricle surface. AT8 channel. Scale bars are indicated in each micrograph

Three-dimensional visual inspection of blocks in the TH channel revealed that NA neurons in the LC core medial part and in the A4 extended dendrites towards the 4th ventricle forming a dense subependymal NA plexus (Fig. 8a–b; video #10, online resource; also see: Fig. 1a–b). A few of these dendrites that derived from LC core pretangle-like cells, were AT8^+^ already in the Braak 0 cases (Fig. 8c–e). The amount of AT8^+^ subependymal processes increased gradually with the Braak stage progression (Fig. 8f–g). Moreover, in the more advanced Braak stages, sparse AT8^+^ processes approached the ventricle surface to 20–50 μm (Fig. 8h).

## Discussion

In the present study, we applied the iDISCO+ volume immunostaining and clearing technology, in combination with LSFM, to explore the cytoarchitecture, tau cytoskeletal pathology and their potential relationships in the human LC/PC region of postmortem brains from subjects afflicted by various stages of the Alzheimer’s disease spectrum. We included 20 subjects from Braak NFT stage 0 till stage 6, with short post-mortem time, lack of α−synuclein co-pathology, and post-mortem perfusion fixation of brains [99], to obtain the highest possible quality standard. This approach allowed a comprehensive, three-dimensional visualization and analysis of not only AT8^+^ cell bodies but also their extensive dendritic arborisations and axons.

### Cytoarchitectural organization of the human LC/PC complex in 3D

To explore the spatial distribution of tau cytoskeletal pathology and investigate the possible spreading of hyperphosphorylated tau within and from the LC, we first studied the cytoarchitecture of LC/PC in Braak 0 subjects, based on TH volume immunostaining. Our 3D analysis revealed frequent ‘duos’ or ‘triplets’ of close ‘hugging’ NA somas in the LC core as well as in all other pericoerulear regions. Recent physiological studies on rodents demonstrate the presence of simultaneously active, yet distinct small LC ensembles with the synchrony of neurons [29, 75, 94]. Moreover, LC neurons with synchronous activity tended to project to functionally related forebrain regions, which may permit a truly targeted neuromodulatory signal from LC [30]. The closely apposed distribution of two, three or rarely even more somas of NA neurons in the human LC may correspond to ensemble-based small functional domains described in rodents. Another remarkable finding based on 3D analysis was the demonstration of dendro-dendritically apposed NA neurons in the human LC/PC region. The presence of dendro-dendritic close appositions [85] or synapses with symmetrical membrane specializations [44] was noted in ultrastructural studies of LC in the rat. Moreover, electric coupling of NA LC/PC neurons via dendro-dendritic gap junctions or via ephaptic interactions were also suggested [15, 31, 49]. Dendro-dendritic synapses, just like in the olfactory bulb [84], form a structural basis for synchronized firing [31], creating a ‘local functional syncytium’ of neurons.

### Large-scale quantification of tau cytoskeletal pathology in the LC/PC complex

We started the characterization of tau cytoskeletal pathology with the quantification of AT8^+^ cells and the full AT8^+^ immunostaining volume in the segmented subregions. The density of hyperphosphorylated tau-filled neurons over the Braak stages is well comparable with earlier data obtained with AT8 antibody staining [10] but lower than calculated based on CP13 antibody staining [35, 37] on sections. According to the present study, while the vast majority of all AT8^+^ neurons is located in the LC core in all Braak stages, there are no significant differences in AT8^+^ cell densities among the subregions, if the AT8^+^ cell number is normalized to the number of TH^+^ neurons. All these findings indicate that the starting point of tau pathology in the LC/PC complex is in fact the LC core. However, the capability of NA neurons to generate and accumulate cytoplasmatic AT8 immunoreactivity is similar in all subregions of the LC/PC complex, and the high number of AT8^+^ neurons in the LC core is rather the consequence of the higher NA cell density in this area.

The subcoeruleus (or sublaterodorsal tegmental nucleus) of the LC/PC complex is involved in the regulation of REM sleep behaviour [83]. Several experimental animal model studies suggest that α−synuclein pathology in the subcoeruleus contributes to the pathomechanism of REM sleep behavioural disorder (RBD) [34, 83, 89]. Importantly, in clinical practice, RBD is thought to be a marker of α−synucleopathy [52, 64]. Indeed, α−synuclein pathology was described in the human LC [33, 93] and ‘coeruleus-subcoeruleus area’ [19, 25] of Parkinson’s disease subjects; however, we are not aware of systematic quantitative studies concerning the spatiotemporal distribution of synuclein pathology in the human LC/PC subregions. The current subject cohort is lack of α−synuclein pathology in the LC/PC region but exhibits tau pathology in the subcoeruleus. Considering the close association of RBD and α−synuclein, but not tau, pathology in the subcoeruleus, we theorize that tau and α−synuclein pathologies affect different cell populations in the subcoeruleus. Due to their clinical relevance, these aspects merit further studies.

### Cellular pathology in the LC/PC complex in 3D and functional implications

Higher resolution 3D scans revealed an intriguingly high morphological heterogeneity of AT8^+^ cellular structures throughout the Braak stages, overall strongly supporting the notion that NFTs are not static but rather dynamic neuronal lesions [67]. Based on 3D imaging, we determined several categories of AT8^+^ cellular structures and their diverse transition forms, which are supposed to represent various maturation levels of tau cytoskeletal pathology, as proposed in Fig. 4. Morphologically, ‘cells with low-intensity staining’, ‘cells with intact processes and inhomogeneously/homogeneously filled somas’ may correspond to so-called pretangles, while ‘cells with mature tangle-like morphology’ likely are comparable with mature NFTs. ‘Cells with partially atrophic dendrites’ and ‘cells with severely atrophic processes’ may represent transition forms. Moreover, our 3D analysis revealed morphologically pretangle-like neurons with fine, 50–250 μm-long filament-like somatic protrusions. It is noteworthy that these cellular forms are enriched in the Braak 1–2 stages and are virtually absent in the very early Braak 0 and the latest Braak 6 stages. The nature/relevance of these structures is unclear and requires further studies. Also, we describe AT8^+^ neurons that exhibit disintegrating morphology. These structures are rarely found in the LC core from Braak stage 1 (albeit they were noticed in one NFT-stage 0 brain as well) and suggest that a small portion of neurons that accumulate cytoplasmatic hyperphosphorylated tau may disintegrate instead of developing to mature NFTs. The structures defined as neuropil threads in thin histological sections may consist of dendritic debris, swollen axonal fragments, filamentous somatic protrusions, as well as cross-sections of intact dendrites or axons belonging to somas that are not contained within that particular Z plane.

3D imaging suggests that the first morphological sign of degeneration in tangle-bearing neurons is the gradual trimming of the dendritic tree, even preceding the axonal degeneration. Mild dendritic trimming of AT8^+^ neurons is an early process in tau cytoskeletal pathology, which can be sparsely noticed already in four out of the six Braak 0 subjects. Meanwhile, cells with partially atrophic dendrites are the dominating cellular forms in Braak 1–2. Gradual atrophy of the dendritic tree may lead to functional deficits like the insufficient electric coupling of synchronized neurons. Moreover, deficient autoinhibition via somatodendritic α2 autoreceptors [65] may result in hyperexcitability of tangle-bearing neurons with a trimmed dendritic tree but still intact axons. All these may contribute to several prodromal symptoms of AD (like sleep disturbances, anxiety and depression), which are consistent with LC dysfunction [75]. Furthermore, deficient dendritic autoinhibition could advance a vicious cycle of worsening tau pathology [100, 103] and may, at the same time, result in a potentially increasing spreading of tau pathology towards cortical areas. These results are in line with the idea that AD pathology comprises both degenerating neurons and surviving cells with impaired functionality [24].

Our 3D analysis supports the notion that the pathogenesis of hyperphosphorylated tau may simultaneously start in the somatodendritic compartment and the axons. Indeed, we could not identify AT8^+^ axons without an associated cell body in any of the six Braak 0 brains; in fact, all noticed processes in all Braak 0 cases could be traced to an AT8^+^ soma. These results are in concordance with recent studies showing that ectopic somatodendritic localization of hyperphosphorylated tau is a very early process, and both initial neuritic tau (pS369/pS404, ‘IN-tau’, detected by PHF1Ab) and initial somatic tau (pT231, ‘IC-tau’, detected by AT180 Ab) are observed in the same neurons in multiple brain regions already before the formation of AT8^+^ pretangles) [11]. Moreover, the subtoxic concentration of glutamate or Aβ−oligomers can enhance de novo synthesis, translation and hyperphosphorylation of tau in the somatodendritic compartment in vitro [58, 60]. Notably, LC neurons are extensively innervated by glutamatergic/adrenergic and glutamateric/orexinergic fibers derived from the lower brainstem [1] and from the lateral hypothalamus [76], respectively.

### Distribution of tau pathology in the LC along the dorso-ventral axis

Spatial analysis showed that the distribution of tau cytoskeletal pathological forms is not homogenous even within the LC core, but more AT8^+^ neurons and increased AT8^+^ immunosignal volume are found in the dorsal, compared to the ventral LC core segment. These results are in line with the early topographical analysis by Busch et al*.* based on Gallyas silver staining from Braak stage 5 subjects [27]. Moreover, our analysis suggests that tau pathology is more advanced in the dorsal segment not only in terms of AT8^+^ staining volume and cell body numbers but also in the composition of AT8^+^ cellular structures. In addition, the uneven distribution of tangle-bearing neurons and their processes along the dorso-ventral axis of the LC core is independent of pathological stages but, instead, seems to be an inherent property of the human LC, being obvious already at the very early precortical Braak 0 stage, until stage 6. Both earlier HRP-based retrograde tracing in rat [62, 63] and modern viral-genetic tracing [82] or fluorescent bead-mediated retrograde tracing studies on mice [97] have demonstrated that NA neurons in the dorsal portion of LC preferably project to hippocampus and neocortex, while cells in the ventral LC project to cerebellum and medulla. In contrast, viral tracing failed to show such preferential projections along the medio-lateral or rostro-caudal axis of the mouse LC [82]. We are not aware of reports that compare LC projection topography between rodents and human. Assuming that similar projection patterns of LC exist in mammalian species including human, the referred tracing studies together with our results indicate that the dorsal LC with more advanced tau cytoskeletal pathological lesions already in the precortical Braak 0 stage project to regions of the brain that are strongly affected by AD pathology, like the hippocampus or neocortex [59].

### Clustering of AT8^+^ cells

Taking the advantage of 3D imaging, we demonstrated the clustering tendency of AT8^+^ cells in the LC core. Dense (clustered) cells formed duos or minigroups (typically 3–4 cells), which often consisted of immediate neighbouring hyperphosphorylated tau-bearing NA neurons. Moreover, closer inspection occasionally revealed dendro-dendritic close appositions between adjacent dense AT8^+^ cells, already in Braak 0. This observation could be compatible with the notion of cell-to-cell propagation of pathological tau between noradrenergic cells in the LC via dendro-dendritic contacts or synapses. The proposal of trans-neuronal transport of tau pathology is bolstered by in vitro studies [57] and in vivo result in animal models [32]. Moreover, a ‘snapshot’ of trans-synaptic transport of hyperphosphorylated tau was recently also demonstrated by immuno-electronmicroscopy in the aged rhesus monkey brain [72]. Finally, the observed ‘tunnel-like’ contacts between immediate neighbouring AT8^+^ cells resemble tunnelling nanotubes that are also candidates for the trans-neuronal spreading of hyperphosphorylated tau [90, 104]. However, higher resolution imaging studies are needed to ultimately prove this prospect.

### Potential axonal spreading of tau pathology

We provide morphological evidence that hyperphosphorylated tau-filled axons of AT8^+^ NA neurons leave the LC and join to the ascending dorsal NA bundle already in the precortical Braak stage 0. Even if we cannot demonstrate that hyperphosphorylated tau reaches the most distal parts of these axons, the results support the idea that LC could serve as an early source of trans-neuronal spreading of tau pathology to the forebrain [24]. This hypothesis was challenged by recent biochemical results showing the lack of tau seeding activity in LC samples of Braak 0–1 subjects [56]. At the same time, the present 3D imaging study affirms that tau pathological lesions in these earliest Braak stages are still sparse in the LC/PC, which stresses the importance of the design and anatomical accuracy of LC micropunch sampling for biochemical measurements [4, 101]. Another concern about LC being a potential early source of spreading tau pathology towards the transentorhinal cortex is the widespread projections of LC to forebrain areas. In this context, one significant question is: where do those proportionally few AT8^+^ LC neurons project in Braak stages 0 or 0–1? Taking the large axon arborisation of individual LC neurons into account [63], even those few dozens to few hundreds of AT8^+^ neurons in the LC in Braak 0–1 may be effective in propagating pathological tau to the transentorhinal cortex, if those neurons have a preferential projection to this brain region. However, this requires further studies. In addition, besides a potential connectome-based spreading, local neuronal activity may also affect the development of tau pathology in a certain cortical brain region [100].

### The subependymal plexus

Here we describe a dense subependymal NA plexus of processes below the 4th ventricle, originating from the LC neurons. This is in agreement with early studies in rats showing that a subset of LC neurons extends their dendrites to the wall of the 4th ventricle, with a supposed neurosecretory and/or chemosensitive function [39, 46]. Moreover, we show sparse hyperphosphorylated tau-filled subependymal terminal dendritic segments already in Braak 0 and a dense subependymal AT8^+^ network of processes in the more advanced Braak stages. High hyperphosphorylated tau levels are found in the cerebrospinal fluid (CSF) in AD and prodromal AD and it is used as a biomarker [17]. Thus, our results raise the possibility of early transmission of hyperphosphorylated tau between the LC-derived subependymal NA plexus below the 4th ventricle and the CSF.

### Limitations

Our work has several limitations. First, even if we believe that the present study is one of the most comprehensive analysis of tau cytoskeletal pathology in the human LC/PC region so far, we have not included the entire rostro-caudal extension of the LC but only about half of it (6–7 mm of the mid region). Thus, we cannot provide data regarding the full rostro-caudal distribution of tau pathology along the whole LC, and we report immunosignal- and cell densities, which are sensitive to the total nucleus volume and/or the total cell number of the LC. Notably, it was shown that as the Braak stage increases by one unit, the LC volume decreases by 8.4% [92], and total LC neuronal loss can average 63% in AD [42]. We applied the AT8 monoclonal antibody [66] to detect pathological tau lesions, which is perhaps the most widely used hyperphosphorylated tau antibody in neuropathology today, being less sensitive for fixation variances [80] and showing excellent inter-laboratory reproducibility in immunohistochemical studies [8]. However, just like any other tau antibody, it does not detect the full spectrum of NFT maturation: the very early IC/IN tau^+^ cells and the latest ghost tangles are not detected by AT8 [67]. Also, while the high morphological heterogeneity of AT8^+^/TH^+^ cellular structures has been comprehensively described in 3D, our methodical approach does not allow multiplex (immuno)histochemical staining of the same block. Thus, we could not discriminate between ubiquitin/Gallyas-negative pretangle material and the ubiquitin/Gallyas^+^ matured NFTs. Moreover, although AT8 is less sensitive for fixation, variations in fixation quality or postmortem effects may affect the detection of thinner AT8^+^ processes (like long axons or dendro-dendritic close appositions of tangle-bearing neurons). Finally, while several millimetres can be overviewed in 3D by an LSFM scan, its resolution is below the confocal microscope’s resolution. This makes the detailed examination of certain cellular structures limited. However, it is very difficult and laborious to find rare cellular forms in sections, and certain studies like cluster analysis or detection of dendritically apposed neurons are nearly impossible without extensive involvement of the 3rd dimension by large-scale volume imaging. Overall, innovative methodical approaches like combination of confocal and LSFM on the same blocks are needed in the future.

### Concluding remarks

In summary, the present study suggests that the unique LC cytoarchitecture, namely, the densely packed and dendritically extensively interconnected isodendritic neuronal network with long axonal projections [77], makes the human LC an ideal anatomical template for the early and effective trans-neuronal spreading of hyperphosphorylated tau. Some reviews argue that the presence of precortical tau lesions in isodendritic core nuclei is not an early part of the Alzheimer’s pathology spectrum but rather reflects transient tau hyperphosphorylation related to physiological processes [13]. Indeed, homeostatic adaptation of the anatomically complex and widespread NA system repeatedly requires the reorganization of the axonal cytoskeleton in LC neurons, which may imply transient tau hyperhosphorylation [22]. Moreover, enhanced cellular activity stimulated by subtoxic glutamate levels results in de novo somatodendritic tau synthesis and hypephosphorylation [58]. However, if dephosphorylation or protein degradation pathways may fail over the time, even in a very few neurons, hyperphosphorylated tau may become sustained and may spread over adjacent neurons within the LC via dendro-dendritic connections, towards the forebrain via the complex axonal arborisation derived from LC neurons, or towards the 4th ventricle via terminal dendritic segments targeting the subependymal NA plexus. Further volume imaging studies of the human brain could comprehensively reveal the temporospatial distribution of tau cytoskeletal pathology in various forebrain areas, in relation to subcortical neuromodulatory systems, including the LC. Accordingly, our present work may be an initial step toward future studies, where state-of-the-art volume imaging, combined with classical histopathological and immunohistological methods as well as with novel biochemical approaches (like seeding activity assays, transcriptomics and proteomics aligned with precise spatial anatomical information), will contribute to new insights in the biology of neurodegenerative brain disorders.

## Supplementary Information

Below is the link to the electronic supplementary material.

Supplementary file1 (MP4 23296 KB)

Supplementary file2 (MP4 23441 KB)

Supplementary file3 (MP4 23174 KB)

Supplementary file4 (MP4 23035 KB)

Supplementary file5 (MP4 22840 KB)

Supplementary file6 (MP4 22666 KB)

Supplementary file7 (MP4 22931 KB)

Supplementary file8 (MP4 22434 KB)

Supplementary file9 (MP4 22905 KB)

Supplementary file10 (MP4 23764 KB)

Supplementary file11 (TXT 22 KB)

Supplementary file12 (TXT 9 KB)

Supplementary file13 (PDF 12443 KB)

## Data Availability

All data needed to evaluate the conclusions in the paper are present in the paper and/or the Supplementary Materials.
