# Supplementary material for: Spatiotemporal characterization of cellular tau pathology in the human locus coeruleus–pericoerulear complex by three-dimensional imaging
Source: Acta Neuropathol. 2022 Aug 30;144(4):651–76. doi: 10.1007/s00401-022-02477-6 (PMC9468059; doi:10.1007/s00401-022-02477-6)
Supplement: Supplementary file 13 — Supplementary file13 (PDF 12443 KB) [file 401_2022_2477_MOESM13_ESM.pdf]

Spatiotemporal characterization of cellular tau pathology in the human locus coeruleus - pericoerulear complex by three-dimensional imaging

SUPPLEMENTARY INFORMATION

Acta Neuropathologica

Abris Gilvesy – Evelina Husen – Zsofia Magloczky – Orsolya Mihaly – Tibor Hortobágyi – Shigeaki Kanatani – Helmut Heinsen – Nicolas Renier – Tomas Hökfelt – Jan Mulder – Mathias Uhlen – Gabor G. Kovacs – Csaba Adori

Correspondence to: Csaba Adori PhD, Department of Neuroscience, Karolinska Institutet, Solnavägen 9, 17177 Stockholm, Sweden. E-mail: csaba.adori@ki.se; adorics@gmail.com. Telephone: +46 7 09983554

SUPPLEMENTARY MATERIALS AND METHODS

Light sheet fluorescence microscopy (LSFM): detailed scanning parameters

Neuromelanin-content of noradrenergic (NA) neurons diffracts the laser that illuminates the focal plane. This is a major obstacle in light sheet microscopy acquisitions of the human locus coeruleus (LC), which we compensated with bilateral illumination of samples scanned in the coronal plane or by scanning the samples in the horizontal plane. Overall, to obtain the required X/Y/Z resolution, homogenous illumination within the entire focal plane and minimal photo-bleaching, each block was routinely scanned with two approaches. More homogenous illumination and moderate file sizes allowed large-scale quantifications after the first method, while higher resolution provided by the second image acquisition pipeline enabled the detailed examination of cellular structures.

1. Scanning with coronal orientation of the LC: 'multicolour acquisition' mode, 2x objective, 2x zoom body and additional magnification of the dipping cap lens (altogether 4.48x effective magnification), 18% and 80% laser powers for AT8 and tyrosine hydroxylase (TH) channels respectively (OBIS 561 and 640 lasers respectively), bilateral illumination ('blend' merging algorithm), 100 ms exposure time, max sheet numerical aperture (0.156), 70% sheet width, 2.0 μm Z-step thickness, no tiling (1.51 μm x 1.51 μm x 2.0 μm voxel size).

2. Scanning with horizontal (dorso-ventral) orientation of the LC: 'multicolour acquisition' mode, 2x objective, 1.25x zoom body and additional magnification of the dipping cap lens (altogether 2.8x effective magnification), 18% and 80% laser powers for AT8 and TH channels respectively (OBIS 561 and 640 lasers respectively), single-side illumination, 100 ms exposure time, max sheet numerical aperture (0.156), 70% sheet width, 2.5 μm Z-step thickness, no tiling (2.42 μm x 2.42 μm x 2.5 μm voxel size).

In addition, selected blocks and selected regions were scanned for TH + AT8 volume co-staining in 'multicolour acquisition' mode with 6.3x zoom body (0.479 μm x 0.479 μm x 2.0 μm voxel dimensions).

3D quantification processes and algorithms applied on LSFM scans

3D segmentation of LC/pericoerulear (PC) complex subregions

LC core and shell, pars cerebellaris (A4) as well as subcoeruleus were 3D delineated and segmented in Imaris 9.2.1. based on anatomical information in 250-500 μm virtual coronal slices, using the manual surface creation function (Supplementary fig. 1) in both the horizontal and the coronal orientations scans. Principles and further details of segmentation are reported in the Results *(3D delineation and segmentation of the human LC/PC complex*). A surface (3D volume) with 4.83 μm grain size was created to cover each subregion, and new TH and AT8 channels were created for each subregion in each scan, while keeping the original voxel dimensions. The volume of each subregion in each block (in μm^3, converted to mm^3) was determined.

Dorso-ventral 3D segmentation of the LC core

The segmented tube-like 'LC core' was further segmented manually to equal-size dorsal and ventral halves, using manual segmentation in 2.5 μm virtual coronal slices in each 250 μm of the horizontal orientation scans. Then, new surfaces with 4.83 μm grain size were created covering the dorsal and ventral segments, and new TH and AT8 channels were created for each segment, with keeping the original voxel dimensions (Supplementary fig. 5a-b). These sub-surfaces and the respective channels were used for further quantifications.

Quantitative determination of TH^+ cell number in LC/PC complex subregions and in LC core segments

In order to reliably quantify TH^+ cell numbers in 3D, a four-step image processing pipeline was created using the ImageJ macro language to subtract local background as well as to balance TH signal intensities in the original scans (raw data). These steps were the following: (i) Adaptive background subtraction and local contrast enhancement. (ii) Images were classified as of low or high intensity, based on their mean pixel intensities. Then, adaptive background subtraction was optimized for the dynamic range of each image. (iii) The TH^+ immunosignal was further segmented based on a mask defined by the 25% highest intensity pixels, which included cell bodies, processes and the scattered light haloes surrounding them. (iv) Finally, based on the mask and a variance cell edge detection, 'unsharpen' function was used to reduce the haloes within the raw images, appropriately separating individual neurons (including neighbouring 'hugging' neurons), as well as enhancing the soma intensity (full script is reported as Supplementary [TH channel image processing Fiji_ImageJ script]; illustration of the process is documented in Supplementary fig. 4a-d).

The processed image stacks were then 3D cropped following the borders of the manually segmented LC/PC subregions. The ultimate identification of TH^+ cells was performed by the 'spot detection' function of Imaris 9.2.1., using 30 μm estimated spot diameter without background subtraction, and 'quality' filter (Supplementary fig. 4e-h). TH^+ cell density (per mm^3 subregion volume), the mean 3D nearest neighbour distance ('spot-to-spot closest distance' MATLAB XTension) and the proportional distribution of TH^+ cells among the subregions was determined.

Regarding LC core segments, scenes of 'dorsal' and 'ventral' core subdivisons were imported into the IMS files with processed LC core Z stacks, the TH channel was masked for the imported surfaces one-by-one, and finally spot detection was applied in the TH channel, in each segment, with exactly the same parameters as used for analysis of the entire LC core. TH^+

cells density (per mm^3 segment volume) and the proportional distribution of TH^+ cells between 'dorsal' vs. 'ventral' segments were determined.

Measuring the length and width of TH^+ cell bodies in the various LC/PC complex subregions

These measurements were applied in 100 randomly selected TH^+ cell bodies in the caudal and rostral ends of the LC core, as well as in 50 randomly selected TH^+ cells from the 'shell', 'A4' and 'subcoeruleus' subregions, in four randomly selected Braak 0 scans with horizontal orientation. The length and width of cells bodies were determined in Imaris 9.2.1 in 2.5 μm-thick planes in 'slice' mode, in the position of the cells when the nucleus was visible with a maximal diameter. Importantly, in each subregion, the spatial orientation of the elongated somas was considered, namely, the 'length' of cell bodies was measured in the anatomically horizontal (X/Y) plane in case of LC core, shell and A4 (in rostro-caudal orientation of cell bodies), while in the anatomically coronal (X/Z) plane in case of the subcoeruleus (in dorso-ventral orientation of cell bodies).

Quantification of the full $AT8^+$ immunosignal volume in LC/PC complex subregions and in LC core segments

The IMS files containing surfaces and TH + AT8 channels for all subregions were downsampled and a new high-resolution surface (3D volume) was created to fully cover the AT8 immunosignal in each subregion (with 2 μm grain size without background subtraction; 'full tau' surface). The full AT8 immunosignal volume was determined as % of the entire subregion volumes. In addition, the full AT8 immunosignal volume was determined in both LC core segments, and its proportional distribution between 'dorsal' vs. 'ventral' segments was determined.

Segmentation and quantification of $AT8^+$ cell body volumes vs. process volumes

In order to segment $AT8^+$ cell body volumes (vs. the volume of processes), the combination of 'volume' and 'oblate ellipticity' filters were applied on the high-resolution

surfaces that covered the total AT8 immunosignal volume. This selection was duplicated as a new surface, which overall covered only the $AT8^+$ cell bodies but not the processes (though short initial dendrite parts were occasionally included in the 'cell body' surface) (Figs. 2a and 3a, h). Subtraction of 'cell body' volume from the 'full AT8 immunosignal volume' resulted in the 'process volume'. The proportion of 'cell body' and 'process' volumes was then determined in each subregion in each scan.

Determination of the number of $AT8^+$ cell bodies in LC/PC complex subregions and in LC core segments

First, surfaces covering the $AT8^+$ cell bodies were created as described in the previous paragraph. Then, 'cell body' surfaces were masked both to AT8 and TH immunochannels, and 'spot detection' function of Imaris 9.2.1. was applied in the AT8 channel, using 30 μm estimated spot diameter without background subtraction, and 'quality' filter. Visual comparison of the newly masked AT8 and TH channels showed that virtually all $AT8^+$ cells were also TH^+ in the examined LC/PC subregions. The number of $AT8^+$ cells (number of spots) was determined and was normalized for segment volume ($AT8^+$ cells / mm^3 volume) and for TH^+ cell number ($AT8^+$ cell number as percentage of TH^+ cell number) in each segment. In addition, $AT8^+$ cells were counted in all LC core segments and their proportional distribution between 'dorsal' vs. 'ventral' segments was determined, with normalization both for segment volume and TH^+ cell number.

Quantitative characterization of $AT8^+$ cellular structures

In order to quantitatively characterize the $AT8^+$ cellular structures, 15 cells were randomly selected in each category and 2 μm grain size 3D-volumes ('surfaces' in Imaris software) were created to cover (i) only cell bodies or (ii) full cell volumes including cell bodies and all traceable processes. Based on these volumes, several three-dimensional quantitative parameters were determined and statistically evaluated (Supplementary fig. 10). The

descriptions below were created with the application of the Imaris software reference manual (http://www.bitplane.com/download/manuals/ReferenceManual9_2_0.pdf). Illustrations and equations provided below are all reported in the same manual.

1./ Oblate and prolate ellipticity of cell body. These numerical parameters reflect for the shape of the somas, as follows:

Ellipsoid (spheroid) is a type of quadric that is a higher dimensional analogue of an Ellipse. Illustration and equation of a standard Ellipsoid in an x-y-z Cartesian coordinate system are:

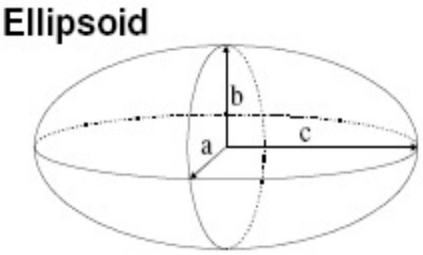

$$\frac{x^2}{a^2} + \frac{y^2}{b^2} + \frac{z^2}{c^2} = 1$$

If a = b < c, it is a prolate spheroid (cigar-shaped); if a < b =c, it is an oblate spheroid (disc-shaped):

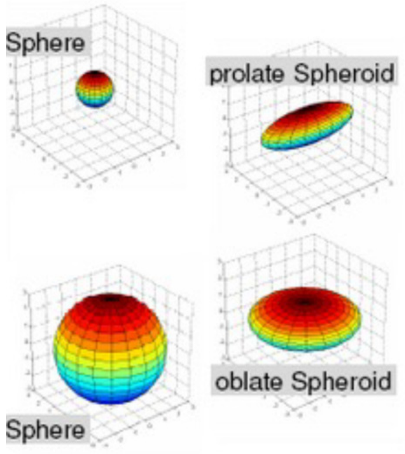

Accordingly, formulas to calculate oblate ellipticity (e_{oblate}) and prolate ellipticity (e_{prolate}) are the following:

$$e_{oblate} = \frac{2b^2}{b^2 + c^2} \times \left(1 - \frac{2a^2}{b^2 + c^2}\right) \qquad e_{prolate} = \frac{2a^2}{a^2 + b^2} \times \left(1 - \frac{a^2 + b^2}{2c^2}\right)$$

2./ Sphericity of cell body (Ψ). This numerical parameter reflects for the shape of the soma. Sphericity is a measure of how spherical an object is. It is the ratio of the surface area of a sphere (with the same volume as the given particle) to the surface area of the particle:

$$\psi = \frac{\pi^{\frac{1}{3}}(6V_p)^{\frac{2}{3}}}{A_p}$$

(V_p = volume of the particle; A_p = surface area of the particle.)

The sphericity of an ideal sphere is 1.000. The closer the value of Ψ is to 1, the nearer the shape of the object to an ideal sphere becomes.

3./ Immunostaining intensity of cell body. The sum of the voxel intensities that are enclosed the 3D volume of the segmented cell body.

4./ Cell body volume in μm^3.

5./ Full cell surface area in μm^2. The surface area of the entire cell, including all traceable processes (axon and dendrites).

6./ Full cell volume in μm^3. The volume of the entire cell, including the cell body and all traceable processes (axon and dendrites).

7./ Process volume in μm^3. The volume of all traceable processes (axon and dendrites).

8./ Number of dendritic branches in one cell.

Spatial distribution of AT8$^+$ processes in proximity of AT8$^+$ cell bodies

Downsampled IMS files with AT8$^+$ cell body detection (spots) and ‘full tau’ surface (covering the full AT8$^+$ immunosignal volume) were opened in Imaris 9.7.2., and the ‘object-object statistics’ function was activated both for the spots (representing the AT8$^+$ cell bodies) and the surfaces (representing the full tau volumes). Then, the filter ‘shortest 3D distance to spots’ was applied on the ‘full tau’ surfaces with 50-150 µm, 150-250 µm, 250-350 µm, 350-450 µm, 450-550 µm, 550-650 µm and 650-750 µm ranges. All these selections were then

duplicated as new surfaces and were colour-coded in case for each subregion. These new surfaces divided the AT8$^+$ process volume to concentric sphere-like spatial zones around the cell bodies (the 0-50 μm spatial range was excluded, since this contained the actual cell body volumes). Surface volumes (μm^3) in all spatial ranges were determined, and the proportion of tau process volumes in these spatial zones was calculated as percentage of the full tau process volume. In case of the shell, A4 and subcoeruleus, Braak 0 blocks without detected AT8$^+$ cell bodies were excluded.

Spatial distribution analysis of AT8$^+$ cells I: calculation of 'nearest neighbour index' (NNI) in the LC core

LC core scans with AT8$^+$ cell body detection were opened in Imaris 9.2.1. X/Y/Z coordinates of spots representing the geometrical centre of AT8$^+$ cell bodies were extracted, and NNI was calculated for each dataset in MATLAB (full script is reported as Supplementary [NNI calculation MATLAB script]. NNI is an estimate of spatial distribution and indicates whether a point pattern is dispersed (NNI > 1), or clustered (NNI < 1) [1]. NNI was calculated as the ratio of the 'actual (real) average nearest-neighbour distance' to a 'simulated average nearest-neighbour' distance. The 'simulated average nearest-neighbour distance' was calculated by randomly distributing the same number of cells as the actual cell population over the same 3D reference frame (Monte Carlo simulation), followed by calculating the average nearest-neighbour distance based on 1000 repeated simulations [2]. *Paired Student's t-test* was applied for the evaluation of simulated vs. actual nearest neighbour distances.

Spatial distribution analysis of AT8$^+$ cells II: determination of 'dense cells', and in-depth cluster analysis in the LC core

LC core scans with AT8 and TH channels as well as with AT8$^+$ and TH$^+$ cell body spot detections were opened in Imaris 9.7.2. The 3D nearest neighbour distance for both AT8$^+$ and

TH$^+$ cells (represented by spots) were determined by the 'spot-to-spot shortest distance' MATLAB XTension.

Then, first, those AT8$^+$ cells that were closer to each other than 75% of the average nearest neighbour distance were segmented. These cells were defined as '*dense AT8$^+$ cells'*. Then, 'dense cells' were colour-coded and their spatial distribution was further explored and characterized by the 'split spot' MATLAB XTension. Segmentation and 3D visualization of dense AT8$^+$ cells revealed '*duo cells*' and '*minigroups*' (i.e., 3-9 AT8$^+$ cells where each cell is closer to its nearest neighbour than the 75% of the average nearest neighbour distance). The number of 'duos' and 'minigroups' was determined in each scan and were statistically evaluated by one-way ANOVA.

Second, we determined the subset of those AT8$^+$ cells that are in equal or shorter distance to the nearest neighbour AT8$^+$ cell than *the average nearest neighbour distance of the TH$^+$ cells in the actual scan*. Since virtually all AT8$^+$ cells were also TH$^+$ in the LC core of the examined scans, this subset defines the (statistically) *immediate neighbouring AT8$^+$ NA neurons ('neighbouring cells')*. The number of 'neighbouring cells' per 100 AT8$^+$ cells, as well as the percentage of 'neighbouring cells' among all 'dense cells' were determined and were statistically evaluated by one-way ANOVA.

Tracing long AT8$^+$ axons in Braak stage 0 cases

Long AT8$^+$ axons in scans from the Braak 0 subjects #3 and #5 were traced and 3D reconstructed by the filament tracer module of Imaris 9.2.1., using the 'autopath' algorithm with 40 μm 'starting point diameter' and 5.66 μm 'seeding point diameter'. Cell bodies were defined as starting points of the created filaments.

SUPPLEMENTARY FIGURES AND TABLES

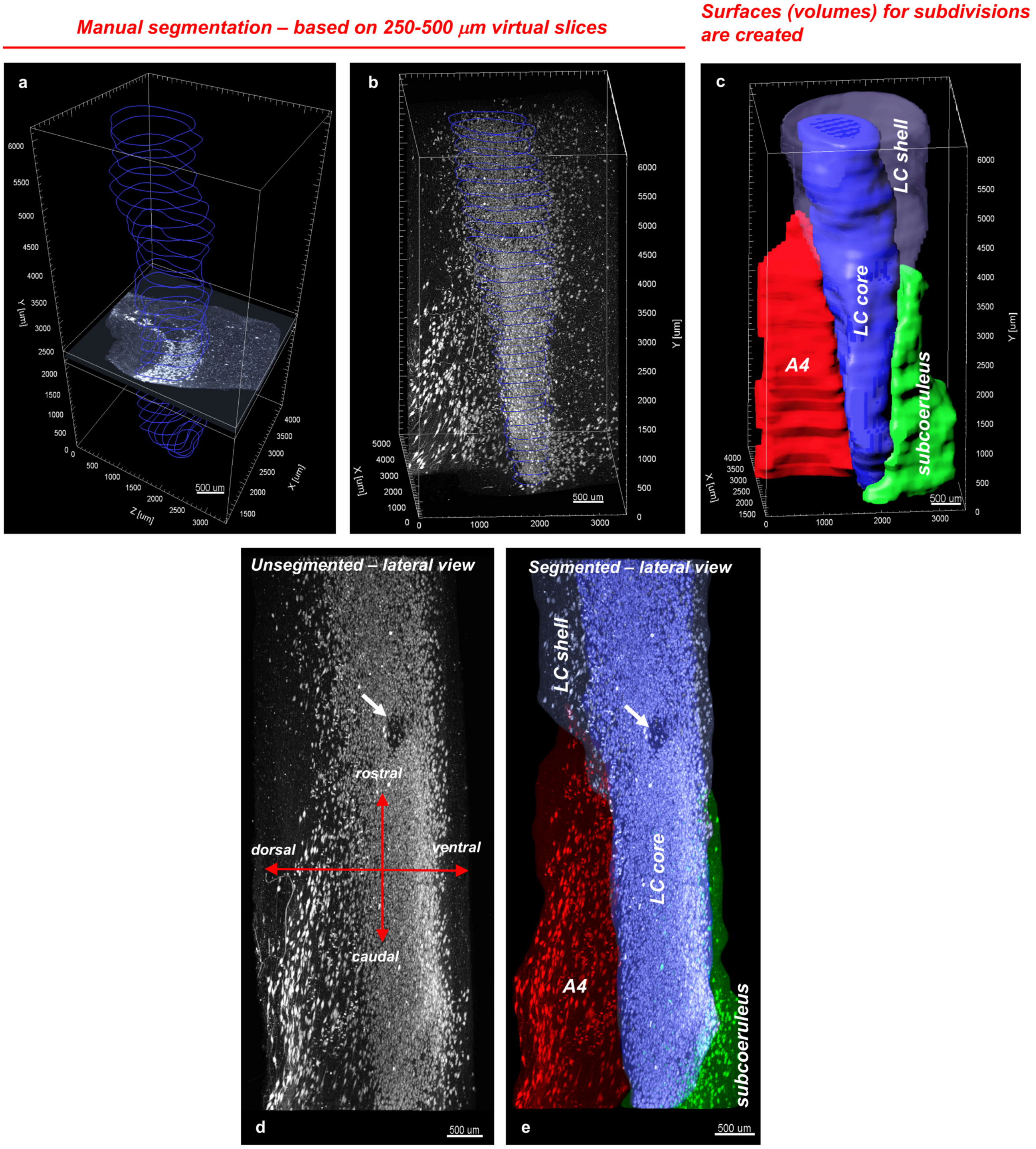

Supplementary fig. 1 Three-dimensional delineation and segmentation of the LC/PC complex.

Subregions were manually segmented based on cell densities and soma orientations, using 250-500 µm-thick coronal optical sections with TH immunochannel. **(a-b)** segmentation of the LC core; blue circles represent the borders of the LC core in every 250 µm. **(c)** 3D reconstructed subregions in a representative block; segmented subregions were handled as separated volumes (surfaces) indicated here by different colours. Within each surface, new channels for AT8 and TH volume staining were created in Imaris. **(d-e)** The same block is shown from lateral view before segmentation (d) and after segmentation (e). Arrows in d and e indicate an artery entering the LC core. Scale bars are indicated in each micrograph.

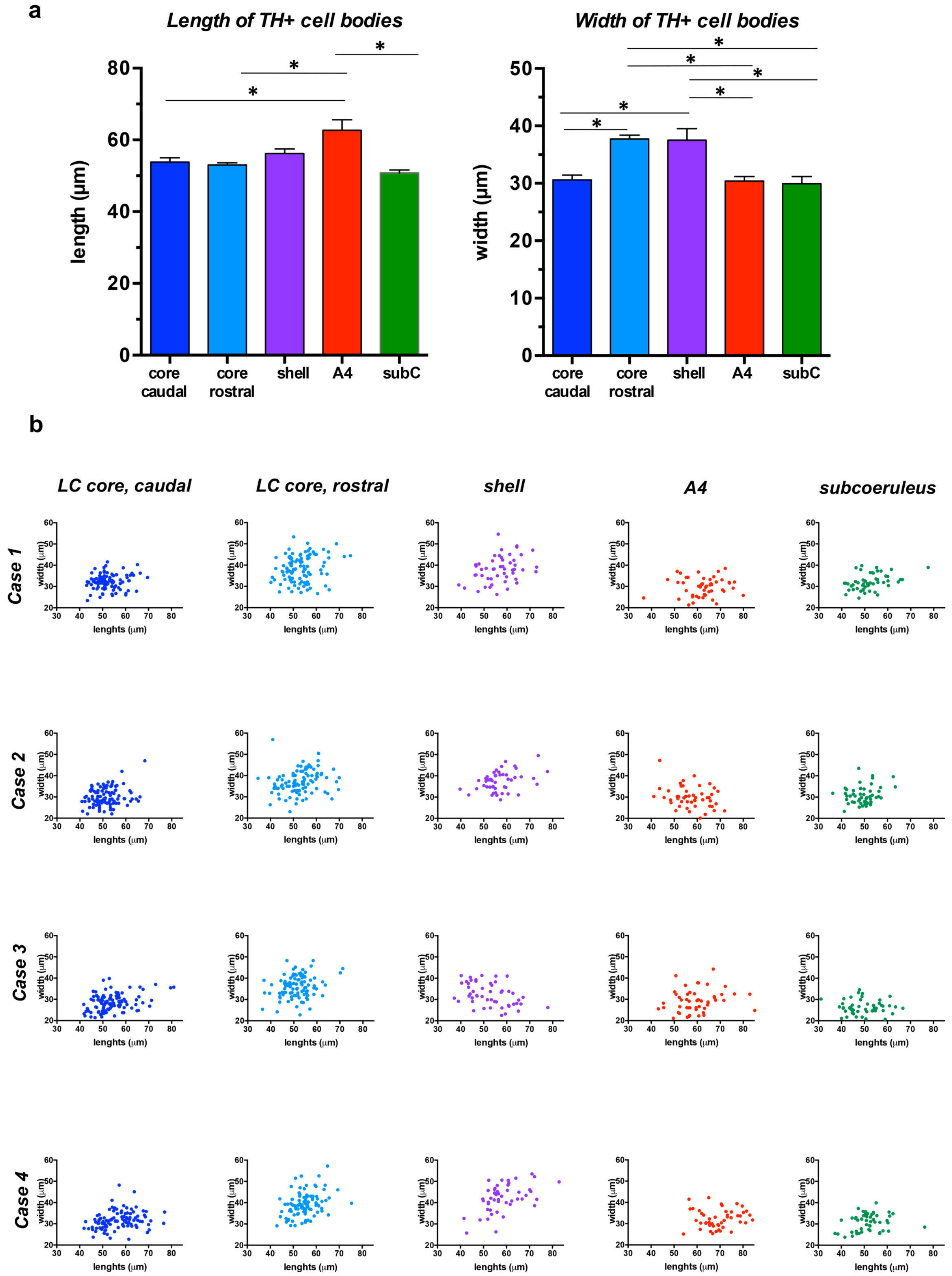

Supplementary fig. 2 Measuring soma sizes of NA neurons in the LC/PC complex.
(a) Average length (a, left panel) and width (a, right panel) of TH^+ cells in the rostral and caudal parts of LC core, in the shell, A4 and subcoeruleus. N = 4 (Braak 0 subjects); *P < 0.05. Data expressed as mean ± SEM. **(b)** Width and length of individual neurons from four Braak 0 subject. In case of LC core, 100-100 cells were counted. In case of shell, A4 and subcoeruleus, 50-50 cells were counted.

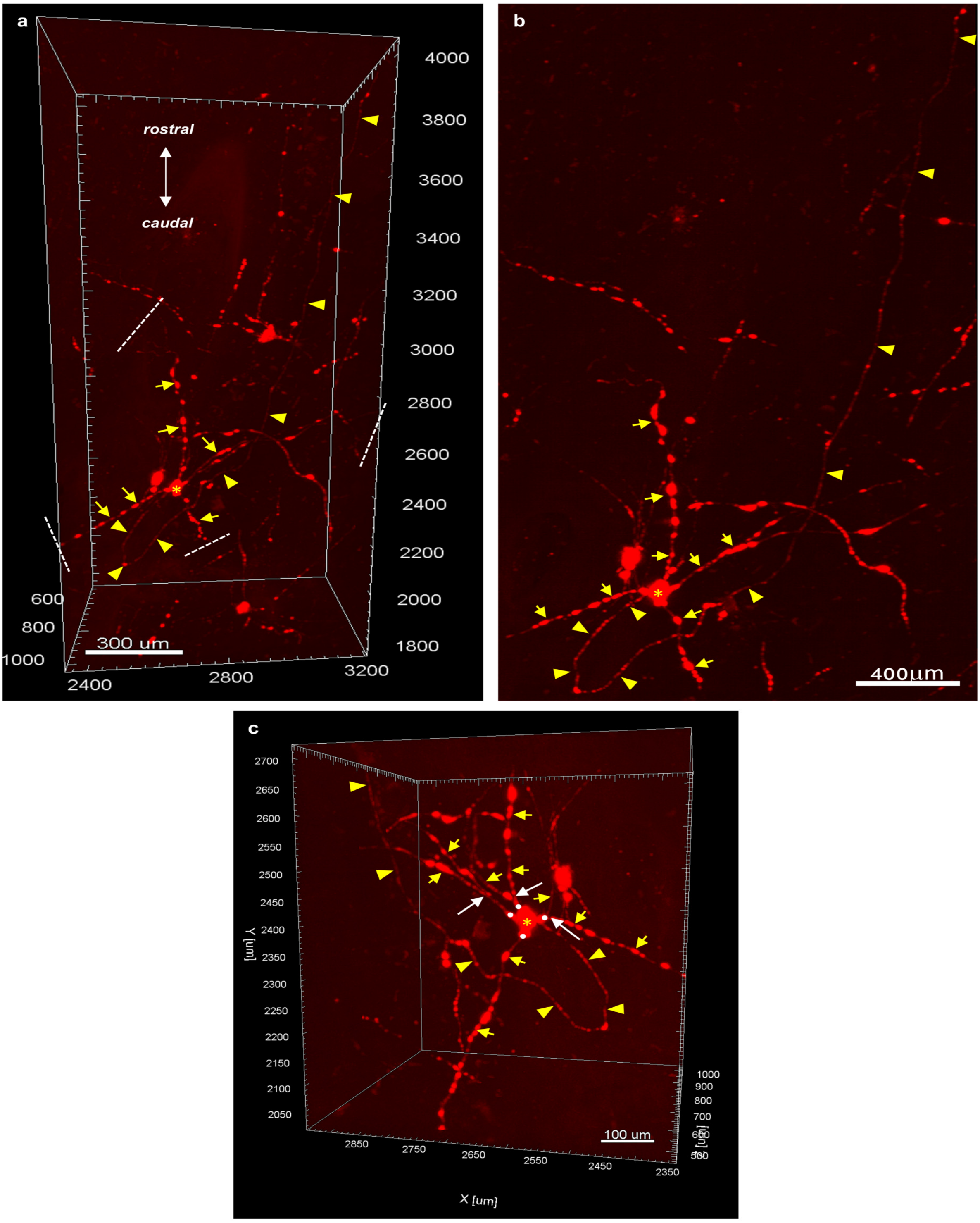

Supplementary fig. 3 Processes of a NA neuron of the LC core (caudal part), visualized by AT8 volume staining from a Braak 0 brain

(a) 3D-rendering of the neuron illustrating the spatial extension of dendritic tree and a ca. 2 mm proximal portion of the axon. *cell body; arrows: dendrite branches; arrowheads: axon. The dashed lines indicate the distal end of dendrite branches. **(b)** 350 µm-thick 2D optical slice with the same neuron. *cell body; arrows: dendrite branches; arrowheads: axon. **(c)** Zoom to the perisomatic region of the same neuron. *cell body; white dots: initiation point of dendrites; yellow arrows: dendrite branches; white arrows: branching point of dendrites; arrowheads: axon. Scale bar is indicated in each micrograph.

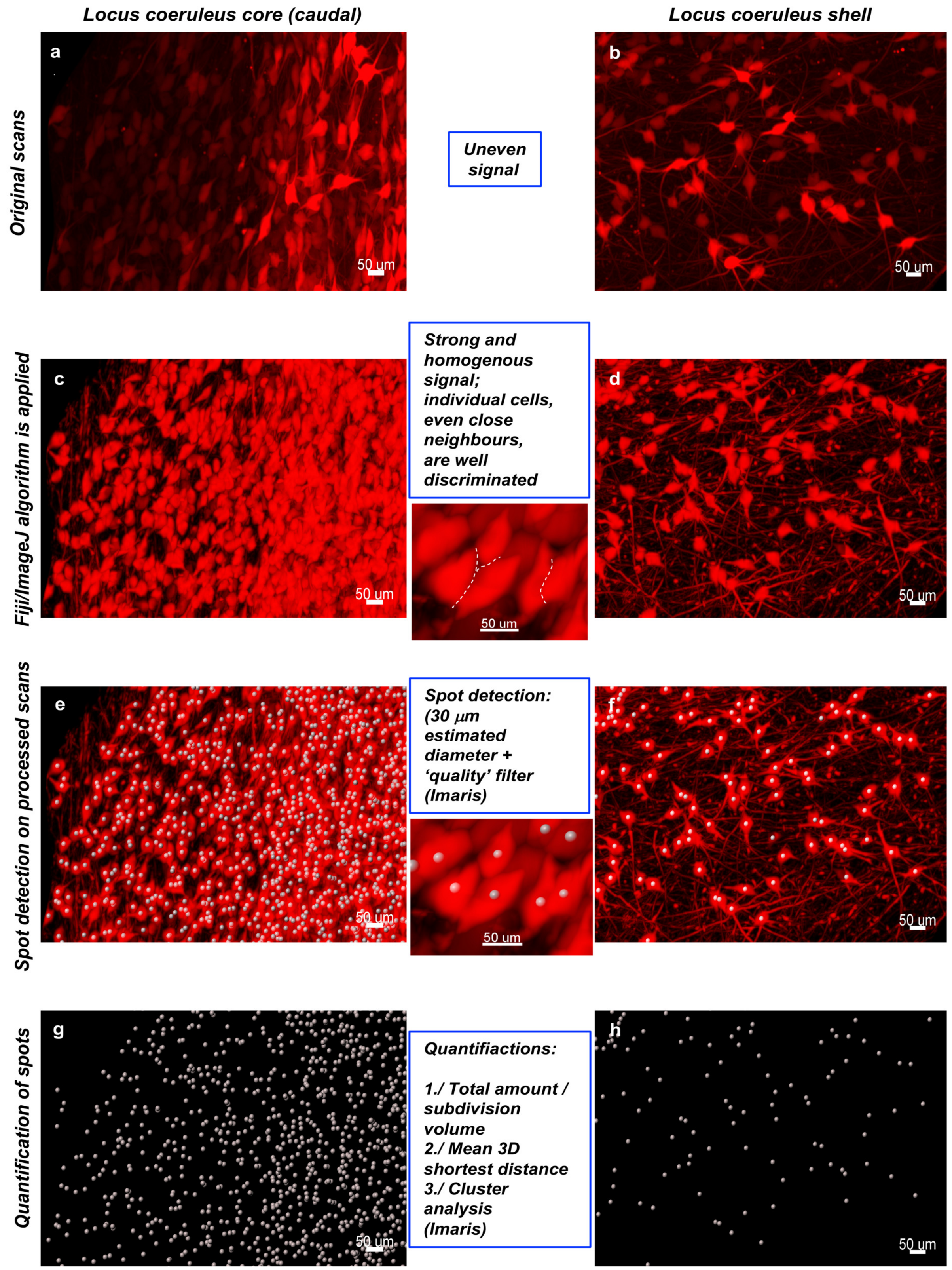

Supplementary fig. 4 Detection pipeline for TH$^+$ neurons.

Representative micrographs (2.5 µm-thin optical slice) are shown from the LC core (a, c, e and g) and from the LC shell (b, d, f and h). **(a-b)** Raw data (original scans). **(c-d)** images processed by a custom-made Fiji/ImageJ macro language script. **(e-f)** Imaris spot detection is applied on the processed images: each TH$^+$ cell is indicated by a spot. **(g-h)** 3D spot cloud was applied for further quantitative analyses. Scale bars are indicated in each micrograph.

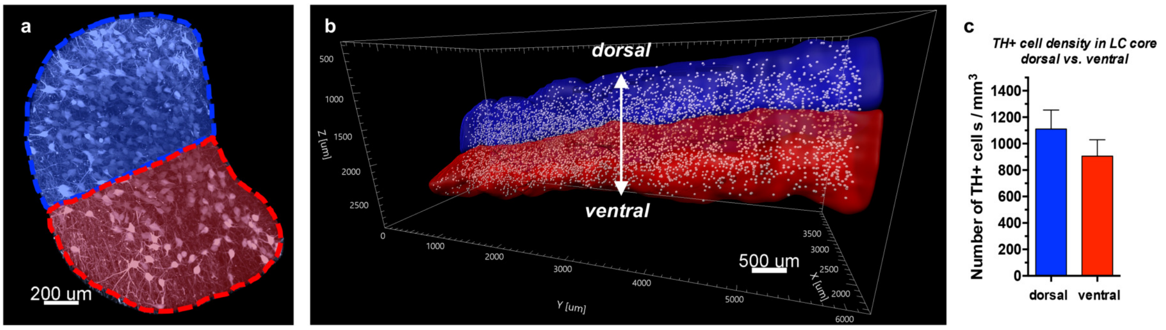

Supplementary fig. 5 Further segmentation of the LC core to equal-size dorsal/ventral halves. **(a-b)** Dorsal (blue) and ventral (red) segments of the LC core shown in a 250 µm thick coronal optical slice with TH volume immunostaining (a) and after 3D reconstruction with spot detection of TH^+ cells (b). **(c)** Quantification of TH^+ cells in the dorsal/ventral parts, normalized for segment volume. N = 8 (Braak 0-2 subjects). Data expressed as mean ± SEM. Scale bars are indicated in each micrograph.

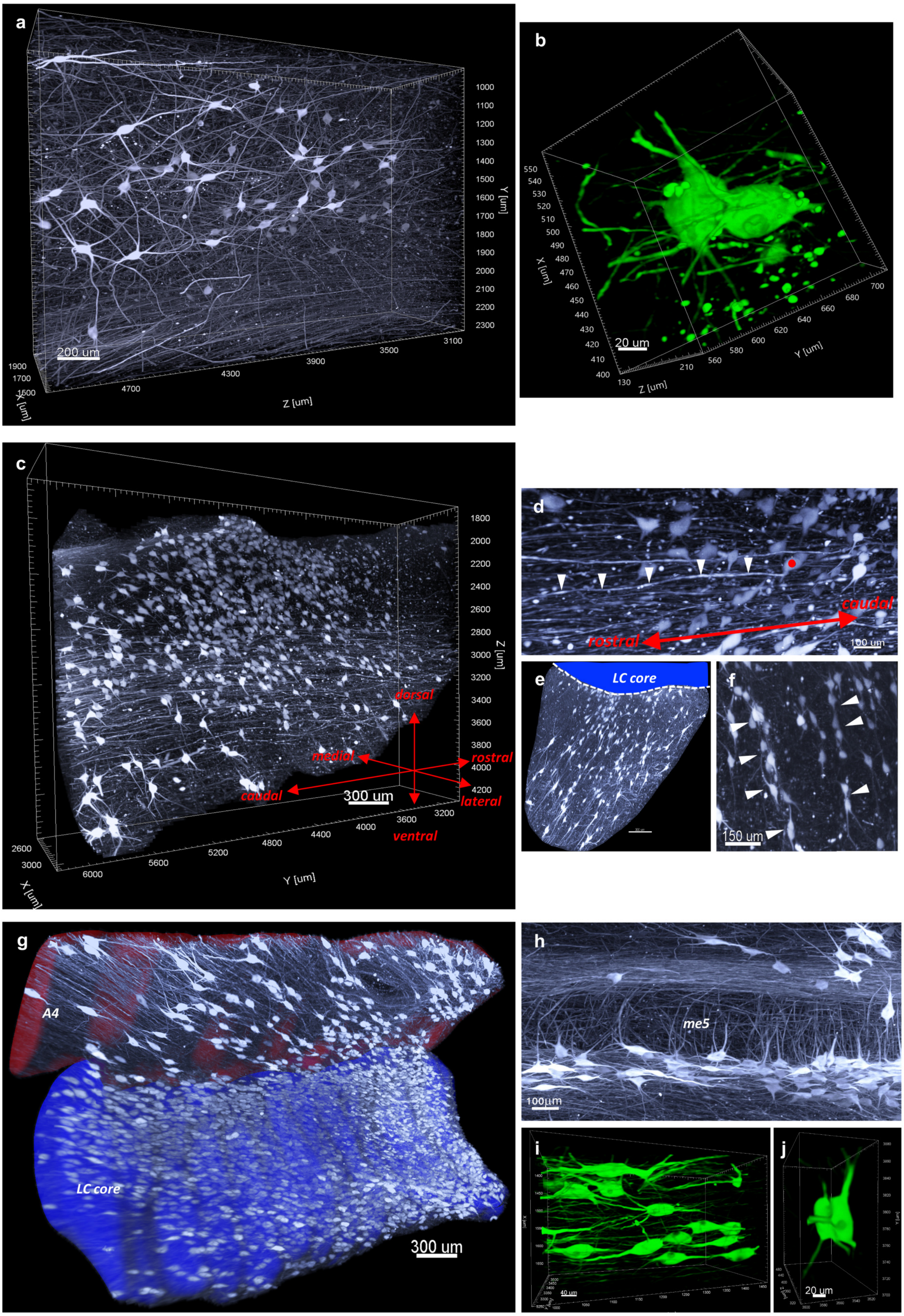
a
1000
1100
1200
1300
1400
1500
1600
1700
1800
1900
2000
2100
2200
2300
Y [um]
200 um
1900
1700
1600
X [um]
4700
4300
3900
3500
3100
Z [um]
b
550
540
530
520
510
500
490
480
470
460
450
440
430
420
410
400
X [um]
20 um
130
210
Z [um]
560
580
600
620
640
660
680
700
Y [um]
c
1800
2000
2200
2400
2600
2800
3000
3200
3400
3600
3800
4000
4200
Z [um]
dorsal
medial
rostral
caudal
lateral
ventral
300 um
2600
3000
X [um]
6000
5600
5200
4800
4400
4000
3600
3200
Y [um]
d
caudal
rostral
100 um
e
LC core
f
150 um
g
A4
LC core
300 um
h
me5
100µm
i
40 um
j
20 um

Supplementary fig. 6 Further exploration of the human LC/PC complex 3D cytoarchitecture. All micrographs are from TH volume immunostaining of Braak 0 brains. **(a-b)** Demonstration of the LC shell. Representative 3D overview of NA neurons in the LC shell (a). 'Hugging' NA neurons from the LC shell (blend 3D rendering mode) (b). **(c-f)** Demonstration of subcoeruleus. Subcoeruleus NA neurons are intermingled with long TH^+ axons of the dorsal NA bundle (c). A subcoeruleus NA axon that joins the dorsal NA bundle. Red dot denotes a cell body that sends its axon (arrowheads) in the rostral direction. (d). Dorso-ventral soma orientation of subcoeruleus neurons (250 μm-thick coronal optical slice) (e). Subcoeruleus neurons are often organized in dorso-ventral 'columns' (arrowheads in a 100 μm-thick coronal optical slice) (f). **(g-j)** Demonstration of A4 (pars cerebellaris of the LC). A4 subregion (red surface) is located dorsally from the LC core (blue surface) (g). A4 neuron cell bodies and processes around the mesencephalic trigeminal tract (*me5*), horizontal view (h). Fusiform A4 neurons (blend 3D rendering mode) (i). 'Hugging' A4 neurons, blend 3D rendering mode (j). Scale bars are indicated in each micrograph.

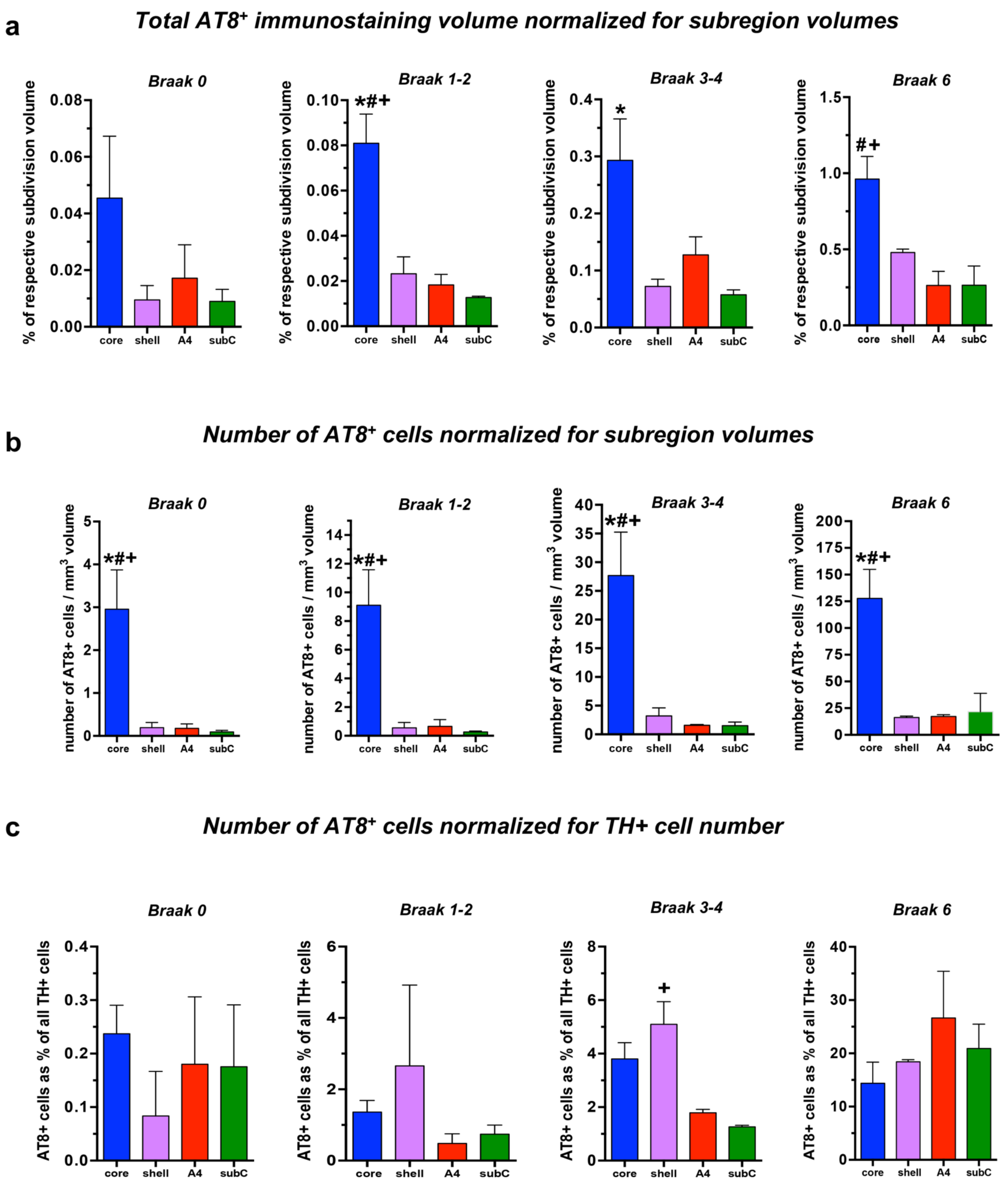

Supplementary fig. 7 Quantitative analysis of the total AT8$^+$ immunostaining volume and AT8$^+$ cell numbers: detailed comparisons between subregions, represented by bar graphs. **(a)** Total AT8$^+$ immunostaining volume normalized for subregion volume. **(b)** Number of AT8$^+$ cells normalized for subregion volume. **(c)** Number of AT8$^+$ cells normalized for TH$^+$ cell number. N = 6 (Braak 0), N = 6 (Braak 1-2), N = 5 (Braak 3-4), N = 3 (Braak 6). *P < 0.05 (vs. shell), $^{\#}$P < 0.05 (vs. A4), $^{+}$P < 0.05 (vs. subcoeruleus). Data expressed as mean $\pm$ SEM.

a

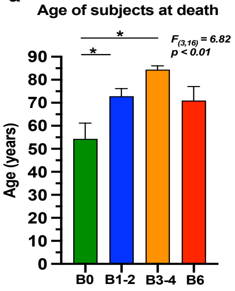

b

Examined parameter	Age covariate				Braak stage factor				Age x Braak stage interaction			
	SumSq	dF	F	p	SumSq	dF	F	p	SumSq	dF	F	p
AT8+ immunostaining volume normalized for subregion volume	0.00048	1	0.024	n.s.	1.84	3	29.93	p<0.0001	0.011	3	0.18	n.s.
Number of AT8+ cells normalized for subregion volume	2	1	0.0051	n.s.	34497	3	24.35	p<0.0001	110	3	0.078	n.s.
Number of AT8+ cells normalized for TH+ cell number	6.11	1	1.738	n.s.	412.14	3	39.12	P<0.0001	56.71	3	5.38	p<0.05

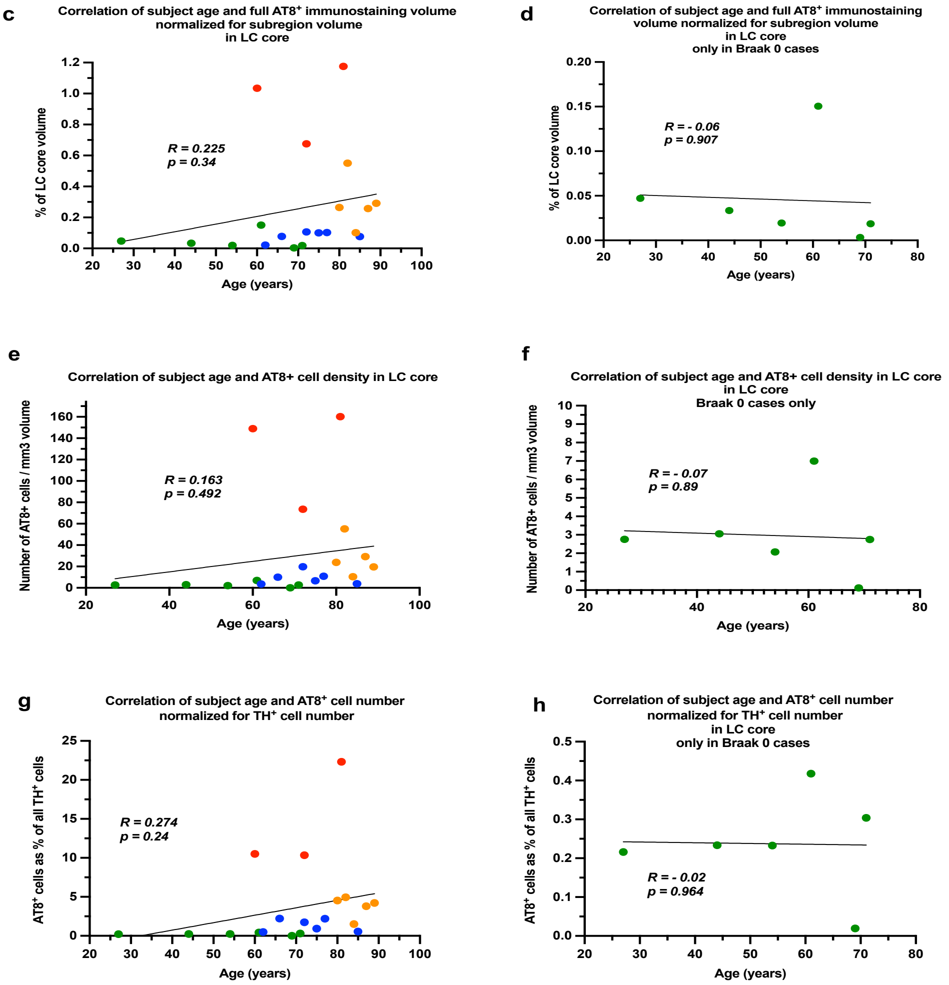

Supplementary fig. 8 Exploring the potential impact of age on the accumulation of tau cytoskeletal pathology in the LC core.

(a) Age of subjects at death. N = 6 (B0), N = 6 (B1-2), N = 5 (B3-4), N = 3 (B6). Data are expressed as mean ± SEM; *P < 0.05. **(b)** Summary table of ANCOVA analysis data. Significant results are represented in red. **(c-h)** Pearson R correlation analyses between the subjects' age at death and (i) the full AT8^{+} immunostaining volume normalized for LC core volume (c-d); (ii) the number of AT8^{+} cells normalized for LC core volume (e-f); as well as (iii) the number of AT8^{+} cells normalized for TH^{+} cell number (g-h). Correlation analyses were performed with the involvement of all subjects from all Braak stages (c, e and g) and with the involvement of Braak 0 subjects only (d, f and h). Green dots: Braak 0 subjects; blue dots: Braak 1-2 subjects; orange dots: Braak 3-4 subjects; red dots: Braak 6 subjects.

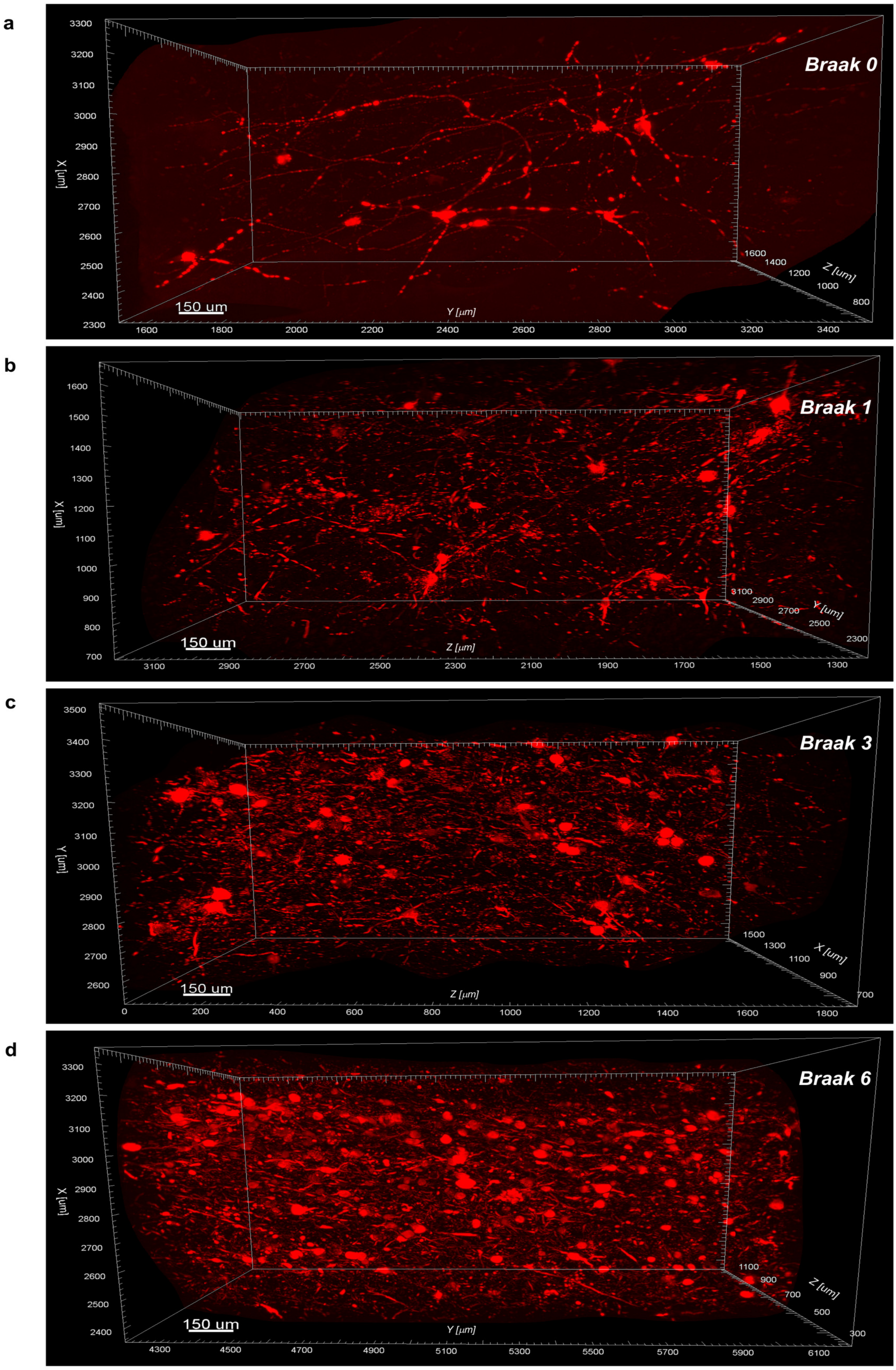

Supplementary fig. 9 Representative overview of AT8 volume immunostaining in the LC core. **(a-d)** 1 x 1 x 2 mm large-scale 3D crops from a Braak 0 (a), a Braak 1 (b), a Braak 3 (c) and a Braak 6 (d) subject. Note the increased density of $AT8^+$ cells and their processes, associated with the more advanced Braak stages. Also note the clustering tendency of $AT8^+$ cells, which is well recognizable in b and c. Scale bars are indicated in each micrograph.

a

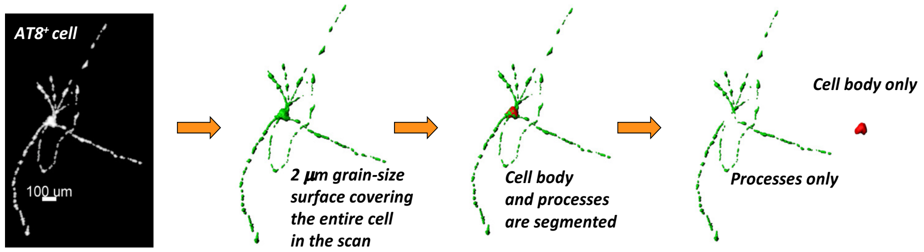

b

AT8+ structure ▶ / *Measured parameter ▼*	*Cell with low intensity staining (low int.)*	*Cell with intact processes and inhomogeneously filled somas (inhomo. filled)*	*Cell with intact processes and strongly and homogeneously filled somas (homo filled)*	*'Stellate-like' cell*	*Cell with fine filamentous somatic protrusions and their perisomatic fragments (perisom. protrus.)*	*Cell with partially atrophic dendrites (partially atroph.)*	*Cell with severely atrophic processes (sever. atroph.)*	*Matured tangle-like cell*
Oblate ellipticity	0.409 ± 0.02	0.378 ± 0.03	0.352 ± 0.02	0.385 ± 0.03	0.302 ± 0.03	0.338 ± 0.02	0.351 ± 0.02	0.334 ± 0.02
Prolate ellipticity	0.444 ± 0.03	0.463 ± 0.04	0.464 ± 0.03	0.361 ± 0.03	0.487 ± 0.03	0.446 ± 0.04	0.478 ± 0.03	0.455 ± 0.04
Spericity	0.777 ± 0.02	0.763 ± 0.02	0.829 ± 0.02	0.854 ± 0.02	0.861 ± 0.02	0.826 ± 0.02	0.848 ± 0.02	0.859 ± 0.02
Cell body staining intensity (arbitrary unit)	683,367 ± 131,761	1,365,733 ± 225,506	3,167,300 ± 609,951	7,634,643 ± 1,854,730	6,293,636 ± 1,303,919	4,956,500 ± 1,352,451	1,493,313 ± 235,627	2,345,357 ± 825,728
Cell body volume (µm3)	70,260 ± 6076	69,693 ± 6025	52,833 ± 10,017	113,427 ± 9899	83,482 ± 7901	67,694 ± 10,447	47,580 ± 5221	46,447 ± 4502
No. of dendrite branches	3.79 ± 0.28	4.00 ± 0.23	4.29 ± 0.22	4.08 ± 0.33	3.70 ± 0.26	3.13 ± 0.30	2.93 ± 0.27	0.00 ± 0.0
Full cell surface (µm2)	33,186 ± 2224	87,050 ± 10158	66,547 ± 4979	46,680 ± 3991	106,227 ± 12,201	47,038 ± 6899	16,958 ± 1151	7292 ± 390
Full cell volume (µm3)	129,957 ± 10,617	325,286 ± 37,329	238,613 ± 25,672	227,307 ± 25,544	350,664 ± 38,129	174,381 ± 24,484	71,225 ± 5814	46,447 ± 4502
Traceable process volume (µm3)	59,697 ± 8615	255,593 ± 36,479	185,780 ± 22,700	113,880 ± 18,622	267,182 ± 37,295	106,688 ± 16,646	23,645 ± 4106	0.00 ± 0.0

c

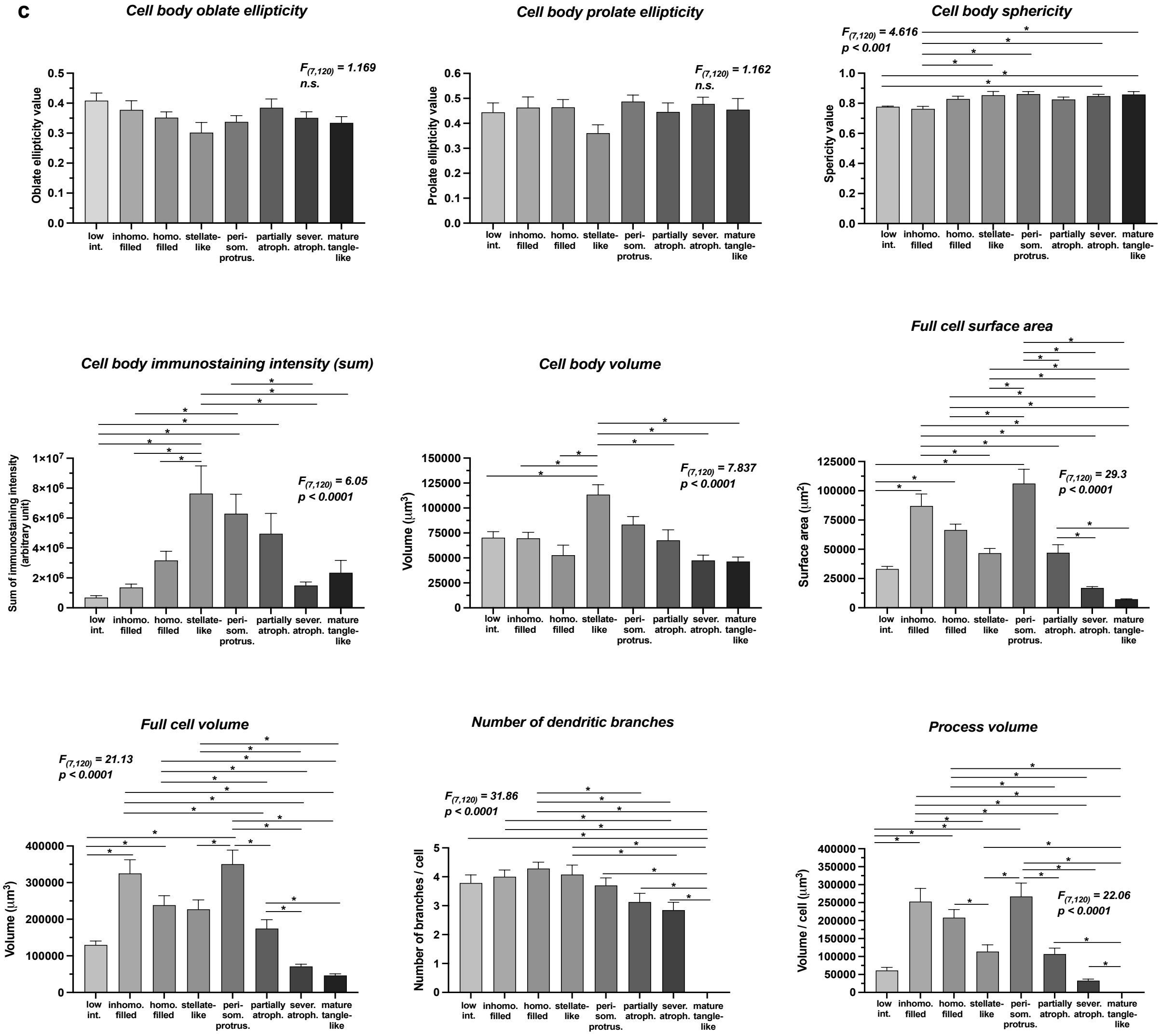

Supplementary fig. 10 Quantitative characterization of AT8⁺ cellular structures.
(a) Segmentation pipeline of $AT8^+$ cells for further quantifications. **(b)** Summary of quantified parameters that characterize $AT8^+$ cellular forms. Dara expressed as mean ± SEM (N = 15 cells in each category). Cellular forms were collected for analysis from Braak 0-1-2 subjects for 'cells with low intensity', 'cells with intact processes and inhomogeneously filled somas, 'cells with intact processes and strongly and homogeneously filled somas' and 'stellate-like cells'; from Braak 1-2 subjects for 'cells with partially atrophic dendrites' and 'cells with fine filamentous somatic protrusions'; from Braak 3-4 subjects for 'cells with severely atrophic processes' and from Braak 6 subjects for 'mature tangle-like cells'. Definitions and explanations of the enlisted parameters are reported in the Supplementary Materials and Methods (*Quantitative characterization of $AT8^+$ cellular structures*). **(c)** Graphical representation and statistical evaluation of the quantified parameters. N = 15 cells / group; data are expressed as mean ± SEM; *P < 0.05.

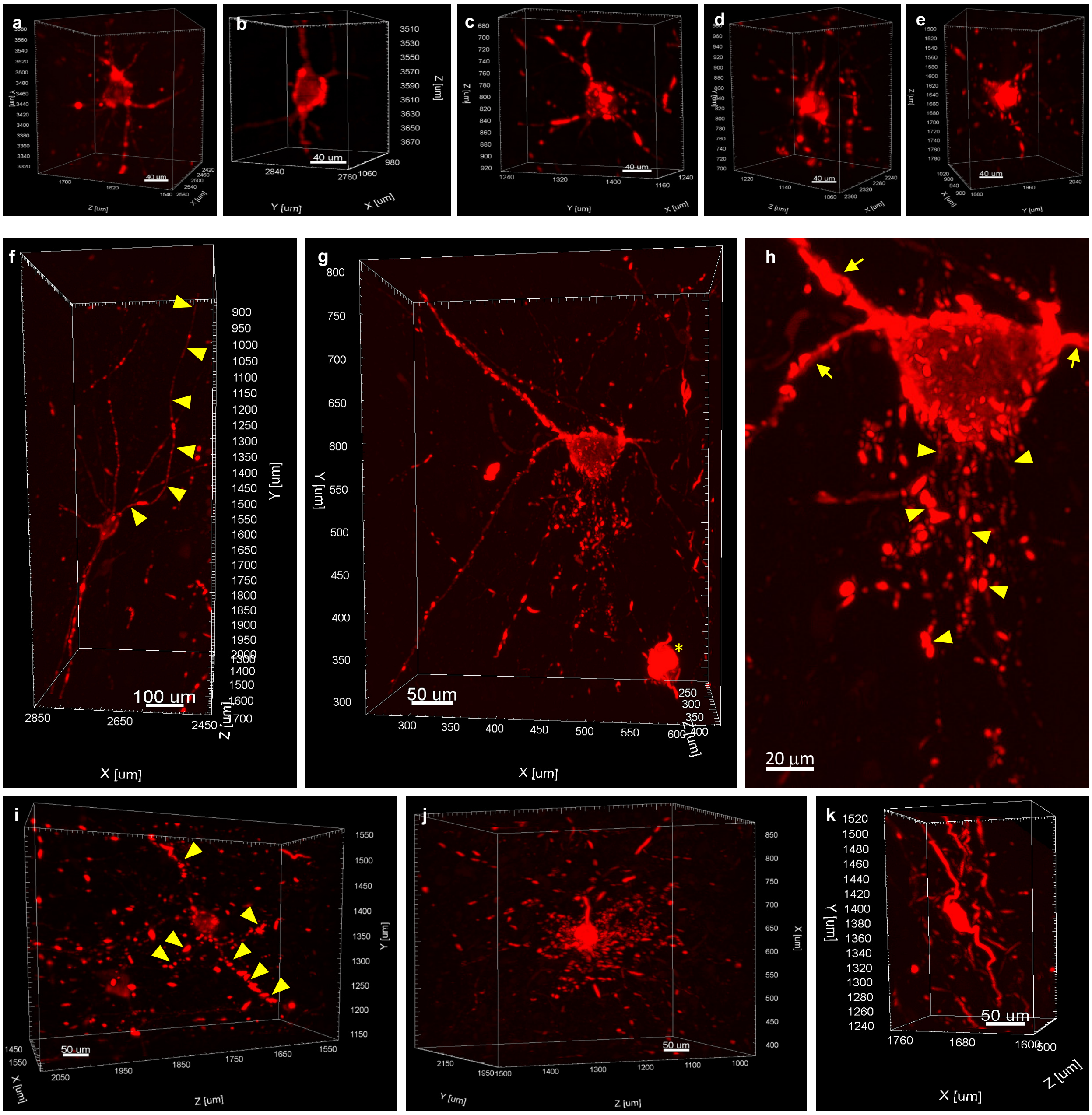

l

AT8+ structure ▷ / Braak stage ▽	*Cells with low intensity staining*	*Cells with intact processes and inhomogeneously-filled somas*	*Cells with intact processes and strongly and homogeneously filled somas*	*'Stellate -like' cells*	*Cells with partially atrophic dendrites*	*Cells with fine filamentous somatic protrusions and their fragments*	*Cells with severely atrophic processes*	*Matured Tangle-like cells*	*Disintegrating cells*	*Long axons*	*Swollen axonal fragments*	*Debris*
**SHELL**												
**B0**	0	0	0	0	0	0	0	0	0	0/+	0	0/+
**B1-2**	0	0/+	0/+	0/+	0	0/+	0	0	0	0/+	0/+	+
**B3-4**	+	0/+	0/+	0	0/+	0	+	0/+	0	0/+	+	++
**B6**	0	0	0	0	0	0	+	++	0	0	++	++
**A4**												
**B0**	0	0	0	0	0	0	0	0	0	0/+	0	0/+
**B1-2**	0/+	0/+	0	0	0/+	0	0	0	0	+	0/+	+
**B3-4**	0/+	+	0/+	0	0	0	0	0	0	+	+	++
**B6**	+	+	0	0	0	0	+	+	0	0	++	++
**SubC**												
**B0**	0	0	0/+	0/+	0	0	+	0	0	+	0	0
**B1-2**	0	0	0/+	0	0	0	+	0	0	+	0/+	+
**B3-4**	0/+	0/+	0/+	0/+	+	0	0/+	0/+	0	+	+	+/++
**B6**	+	+	0/+	0/+	0/+	0	+/++	++	0/+	0	++	++

Supplementary fig. 11 Further demonstration of AT8⁺ cellular structures.
(a-e) Cells with intact processes and inhomogeneously filled somas: variations for the somatic distribution of AT8 immunoreactivity. Strong, granular AT8 staining along the dendrite/axon initial parts (a), along the somatic cell membrane (b), as granules throughout the somatic cytoplasm (c), as large granules in the somatic cytoplasm (d), and as a large compact granule that almost fills up the entire somatic cytoplasm (e). **(f-k)** Proposed transition forms and variations of $AT8^+$ cellular structures. Proposed transition between 'cell with low intensity staining' and 'cell with inhomogeneously filled soma'. Arrowheads: axon (f). Cell with partially fragmented, somatic protrusions (in the middle of the micrograph); *: a mature tangle-like cell (g). The same cell as in the middle of g but as 2D projection at higher magnification. Arrows: dendrites; arrowheads: somatic protrusions (h). Cell with inhomogeneously filled soma and fragmenting dendrites. Arrowheads: fragmenting dendrites (i). Cell with heavily atrophic dendrites and fragmenting somatic protrusions (j). Cell with severely atrophic processes close to a matured tangle-like cell in morphology (k). **(l)** Semiquantitative assessment of $AT8^+$ cellular structures in the pericoerulear area (shell, A4 and subcoeruleus). Scale bars are indicated in each micrograph.

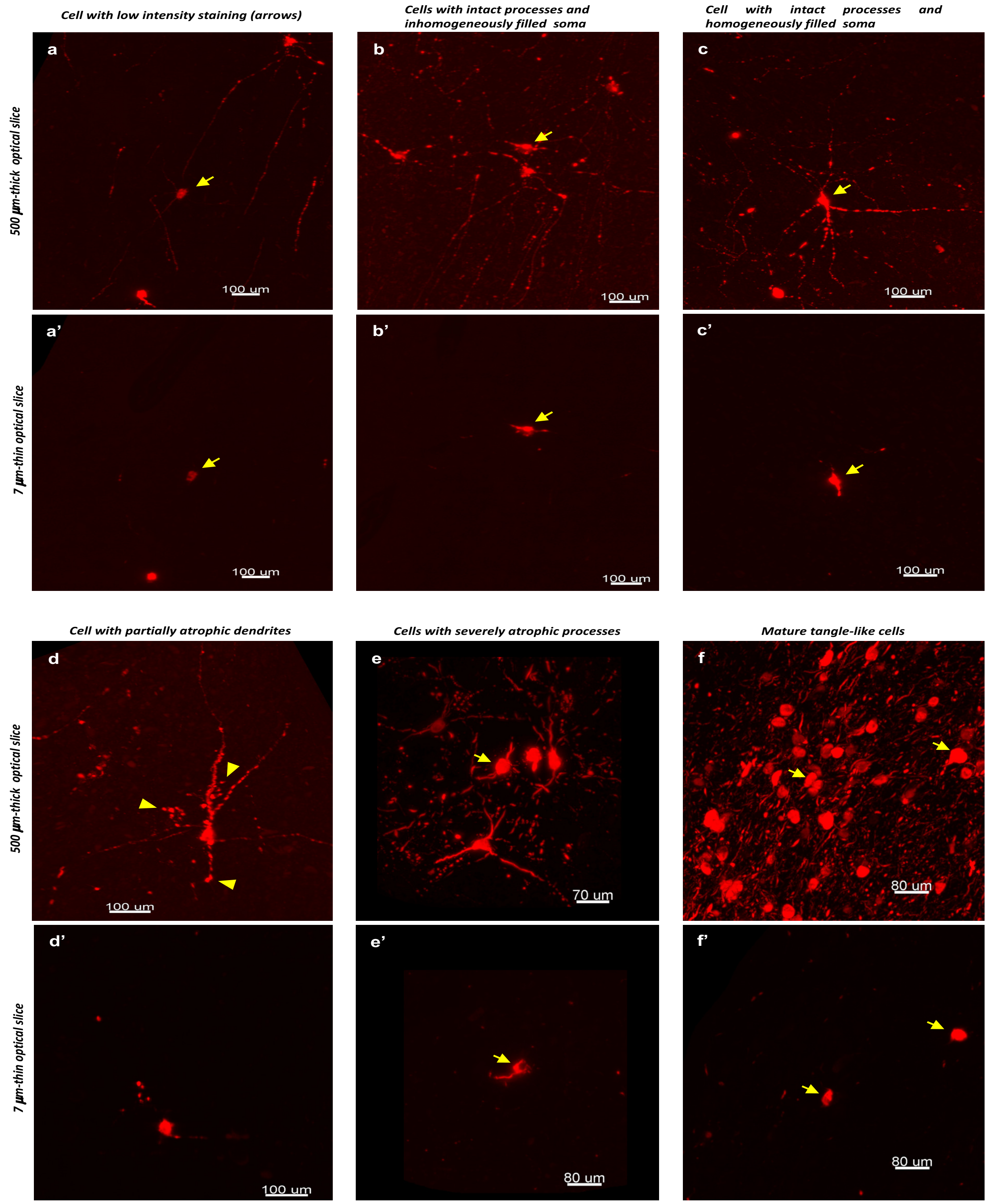

Supplementary fig. 12 Comparison of 2D and 3D representations of the same AT8$^+$ cellular forms 1. **(a-f)** 500 µm-thick optical slices involving entire AT8$^+$ cellular structures with cell bodies and processes. **(a'- f')** 7 µm-thick optical slices representing the same cellular structures as in a - f, focusing on the cell bodies. Note that the evaluation of processes is limited, if possible, in 7 µm-thick optical slices that correspond to routine histopathological sections. Arrows indicate the same cell bodies in the respective 500 µm and 7 µm optical slices; arrowheads in d indicate swollen, fragmenting dendritic branches with degenerating morphology. Scale bars are indicated in each panel.

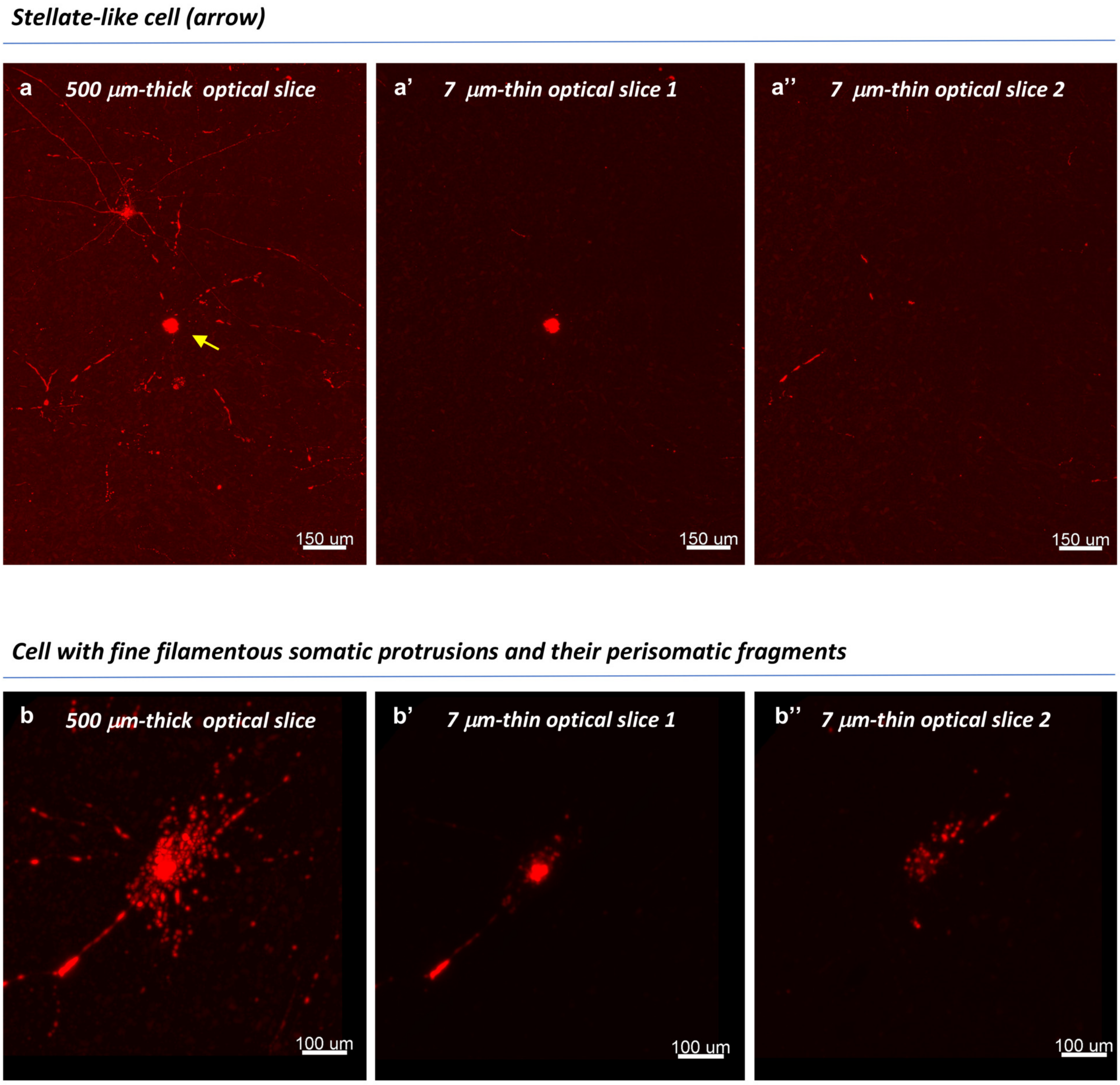

Supplementary fig. 13 Comparison of 2D and 3D representations of the same AT8⁺ cellular forms 2.

(a-b) 500 µm-thick optical slices involving entire $AT8^+$ cellular structures with cell bodies and processes. Stellate-like cell (a) and cell with fine filamentous somatic protrusions and their perisomatic fragments (b). **(a'-a'')** 7 µm-thick optical slices in two different z-levels, representing the same cellular structures as shown in a. These thin optical slices are focusing on the cell body and dendritic processes of the same cell that is indicated with arrow in a (a' and a'', respectively). **(b'-b'')** 7 µm-thick optical slices in two different z-levels, representing the same cellular structures as shown in b. These thin optical slices are focusing on the cell body and the somatic protrusions (b' and b'', respectively). Scale bars are indicated in each panel.

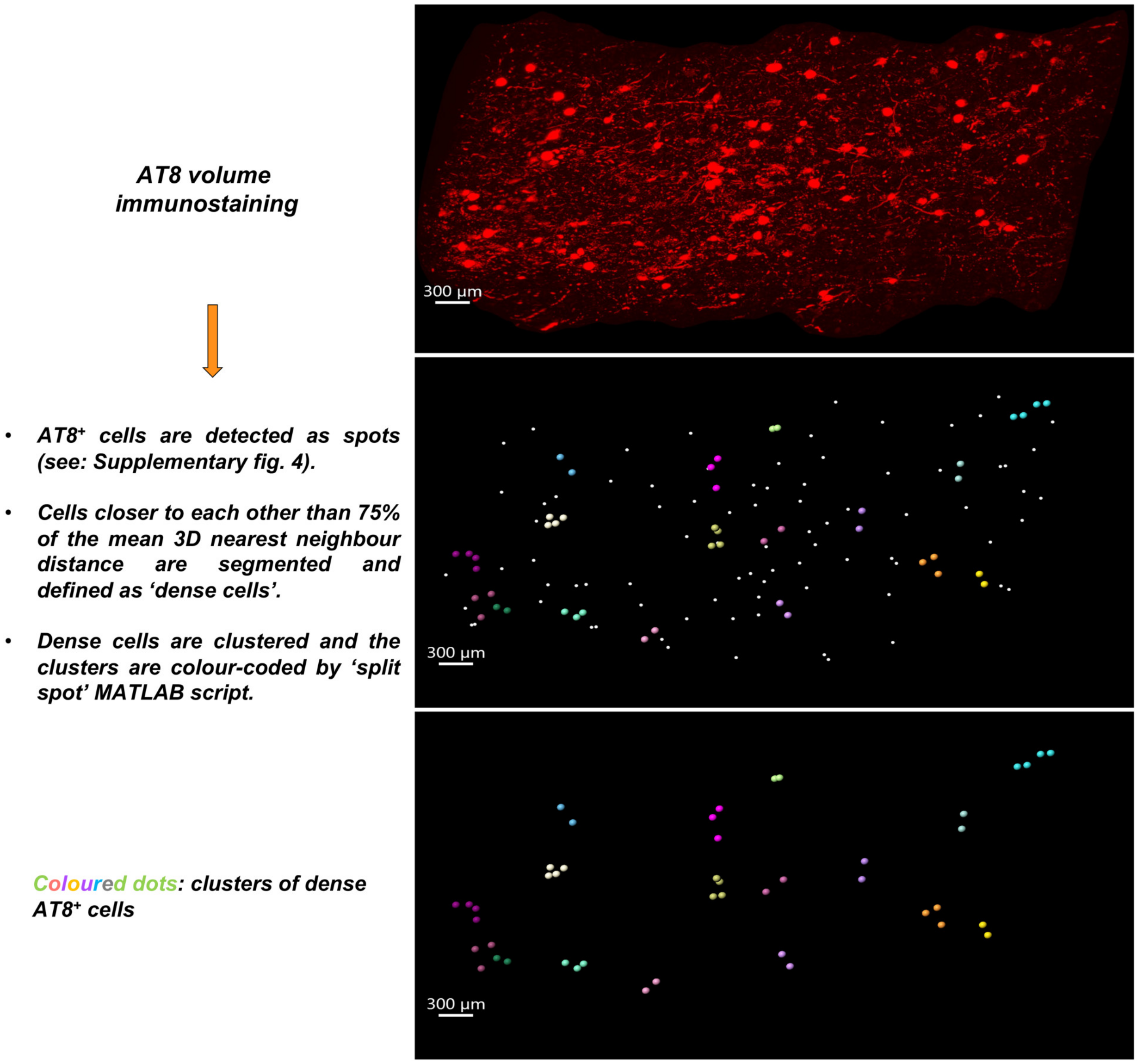

Supplementary fig. 14 Visual explanation for segmentation and clustering of 'dense AT8^{+} cells'.
Scale bars are indicated in each micrograph.

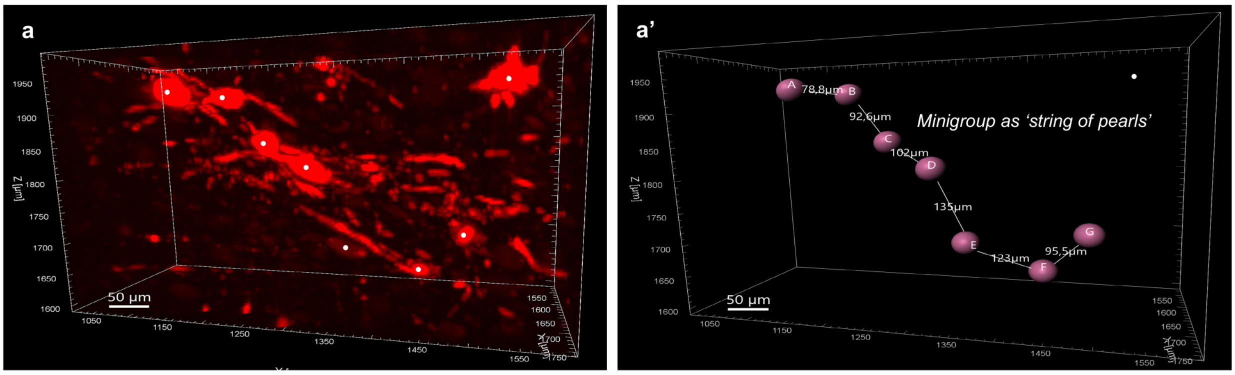

Supplementary fig. 15 Minigroup with the shape of a string of pearls, consisted of seven dense AT8^{+} neurons

Small white dots in panel **a** represent AT8^{+} cell bodies. Coloured spots in panel **a'** refer to the clustered AT8^{+} cells in panel **a**. Scale bars are indicated in each micrograph.

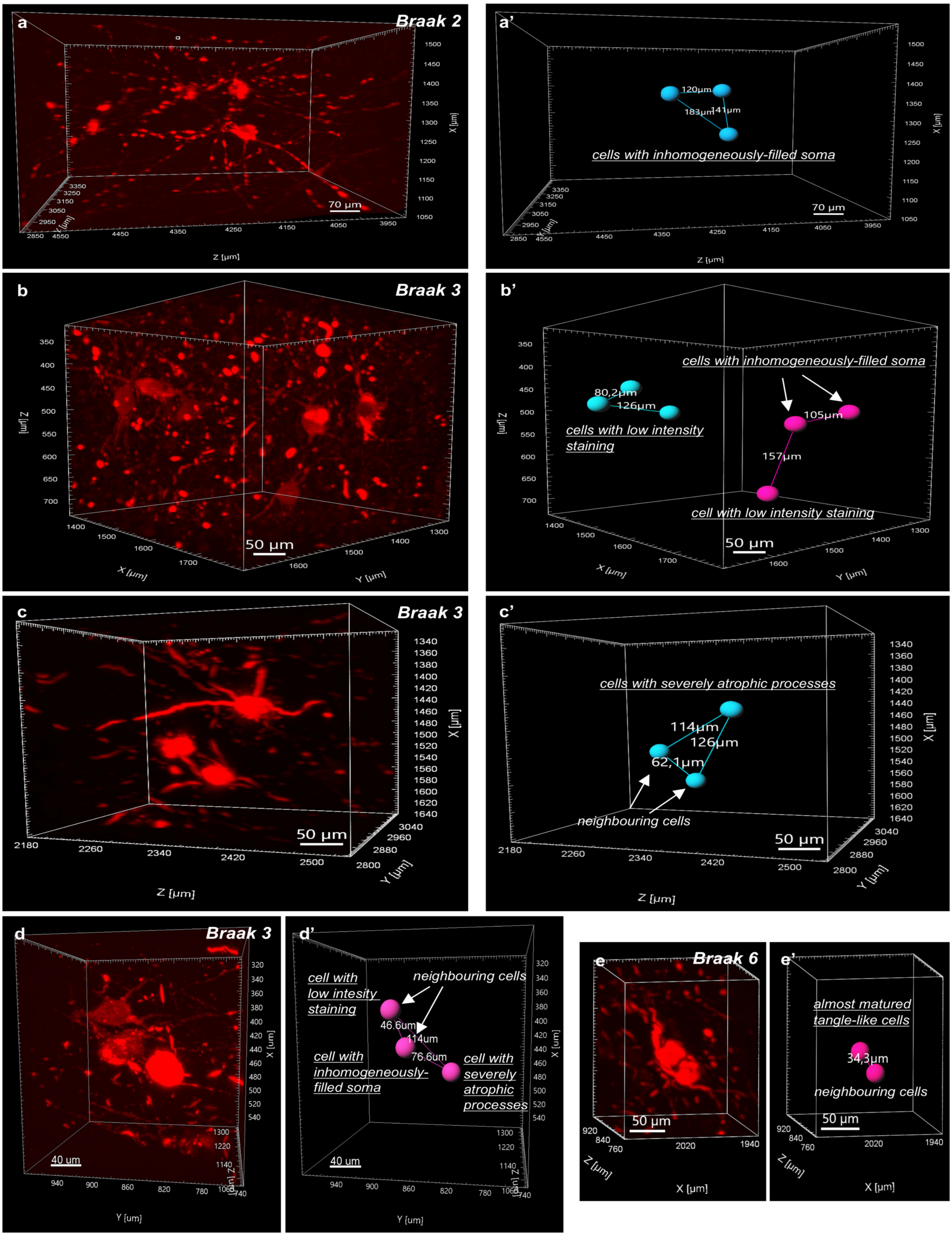

Supplementary fig. 16 Heterogeneity among dense cells.

Coloured spots in a', b', c', d' and e' refer to the $AT8^{+}$ cells in panels a, b, c, d and e, respectively. Scale bars are indicated in each micrograph.

Case no	*Braak NFT stage*	*Age*	*gender*	*PMD*	*Underlying disease*	*Cause of death*
Case 1	0	71y	female	3h	COPD	Respiratory arrest
Case 2	0	61y	female	3h	Subsequent myocardial infarction of inferior wall	Acute transmural infarction of inferior wall
Case 3	0	27y	male	4h	Malignant neoplasm, without specification of site	Pulmonary embolism w/o mention of acute cor pulmonale
Case 4	0	54y	female	3,5h	Chronic ischaemic heart disease	Acute transmural myocardial infarction of inferior wall
Case 5	0	44y	female	8h	None	Acute death (aorta rapture)
Case 6	0	69y	female	9h	Bronchial carcinoma	Bronchopneumonia
Case 7	1	62y	male	14h	Mitral insufficiency	Myocardial infarct; cardiac insufficiency
Case 8	1	72y	male	2,5h	Malignant neoplasm of lung (lower lobe)	Respiratory arrest
Case 9	1	66y	male	3,5h	Dilated cardiomyopathy	Conduction disorder
Case 10	2	85y	male	3h	Calculus of gallbladder with other cholecystitis	Congestive heart failure
Case 11	2	77y	male	5h	Malignant neoplasm of prostate	Heart failure
Case 12	2	75y	female	4,5h	Acute endocarditis	Acute transmural infarction of inferior wall
Case 13	3	84h	male	3h	Acute bronchitis	Respiratory arrest
Case 14	3	80y	female	3h	Acute respiratory failure	Functional intestinal disorder
Case 15	3	82h	female	2h	Old myocardial infarction	Heart failure
Case 16	3	87	female	11h	Chronic cardial decompensation	Respiratory failure
Case 17	4	89y	female	2h	Acute bronchitis	Pneumonia
Case 18	6	72y	female	12h	Alzheimer's disease	Bronchopneumonia
Case 19	6	81y	female	2h	Alzheimer's disease	Bronchopneumonia
Case 20	6	60y	male	6h	Alzheimer's disease	Bronchopneumonia

Supplementary table 1 Basic data, underlying disease and cause of death of the involved subjects.

Case no	*Braak NFT stage*	*Thal-phase (Aβ)*	*CERAD*	*NIA-AA ABC AD criteria*	*α-synuclein*	*TDP-43*	*Vascular lesions*	*PART/ARTAG*	*Other notes*
Case 1	0	0	0	Not AD	negative	negative	no	no	no
Case 2	0	0	0	Not AD	negative	negative	no	no	no
Case 3	0	0	0	Not AD	negative	negative	no	no	no
Case 4	0	0	0	Not AD	negative	negative	SVD	no	no
Case 5	0	0	0	Not AD	negative	negative	no	no	few eosinophilic neurons in hippocampus
Case 6	0	0	0	Not AD	negative	negative	no	no	no
Case 7	1	0	0	Not AD	negative	negative	no	yes	no
Case 8	1	1	0	Low probability	negative	negative	Mild SVD	yes	no
Case 9	1	0	0	Not AD	negative	negative	no	no	no
Case 10	2	1	0	Not AD	negative	negative	Mild SVD	mild ARTAG in medial temporal lobe	Moderate CAA
Case 11	2	0	0	Not AD	negative	negative	no	no	no
Case 12	2	0	0	Not AD	negative	negative	Mild SVD	no	no
Case 13	3	1	0	Low probability	negative	negative	Mild SVD	no	no
Case 14	3	2	1	Low probability	negative	negative	Mild SVD	no	no
Case 15	3	2	0	Low probability	negative	negative	no	no	no
Case 16	3	3	1	Intermediate probability	negative	negative	no	Mild ARTAG in amygdala	no
Case 17	4	0	0	Intermediate probability	negative	negative	no	yes	no
Case 18	6	4	3	Alzheimer's disease	negative	negative	Periventricular white matter microinfarcts	no	no
Case 19	6	4-5	3	Alzheimer's disease	negative	sparse, restricted to hippocampus	Moderate SVD	no	Mild CAA
Case 20	6	5	3	Alzheimer's disease	negative	negative	no	no	CAA type 2

Supplementary table 2 Neuropathological analysis of the applied brains. PART = primary age-related tauopathy; ARTAG = Aging-related tau astrogliopathy; SVD = small vessel disease; CAA = cerebral amyloid angiopathy.

Case numbers and Braak stages	*Cells with low intensity staining*	*Cells with intact processes and inhomogeneously filled somas*	*Cells with intact processes and strongly and homogeneously filled somas*	*'Stellate -like' cells*	*Cells with partially atrophic dendrites*	*Cells with fine filamentous somatic protrusions and their perisomatic fragments*	*Cells with severely atrophic processes*	*Mature tangle-like cells*	*Disintegrating cell*	*Long axons*	*Swollen axon fragments*	*Debris*
#1, Braak0	+	+	+	+	+	0	0	0	0	0	0/+	0/+
#2, Braak0	+	+	+	+	+	0/+	+	0	+	+	0	0/+*
#3, Braak0	+	+	+	0	0/+	0	0	0	0	+	0	0/+
#4, Braak0	0/+	+	+	0	0	0	0	0	0	0	0	0
#5, Braak0	+	+	+	0	0/+	0	0	0	0	+	0	0/+
#6, Braak0	0	0/+	0	0	0	0	0	0	0	0	0	0
#7, Braak1	+	+	+	+	++	+	0	0/+	0/+	0	0	+
#8, Braak1	0	+	+	0	++	0/+	++	+	+	+	+	++
#9, Braak1	+	++	++	+	++	++	+	0	0	+	+	++
#10, Braak2	+	+	+	+	++	+	+	0	0	+	+	++
#11, Braak2	++	0	0	0/+	+	+	++	0	0	0	+	++
#12, Braak2	++	++	++	+	++	0/+	+	0	+	+	+	++
#13, Braak3	+++	+	+	0	+	0	++	+	0	0	++	++
#14, Braak3	+	+	+	0	+	0	+++	+	+	0	++	++
#15, Braak3	+	+	0	0	+	0	+++	++	+	0	++	++
#16, Braak3	+	+	0	0	+	+	++	++	0	+	+	++
#17, Braak4	+	+	+	0	+	0/+	++	+	0	+	++	++
#18, Braak6	+	+	0	0	+	0	++	+++	+	0	++	+++
#19, Braak6	+	+	+	0	+	0	+	+++	+	0	+++	+++
#20, Braak6	+	+	0	0	+	0/+	+	+++	+	0	++	+++

Supplementary table 3 Semiquantitative scoring of $AT8^{+}$ structures in the LC core, indicated for all examined subjects in details. The summary of this table is reported as Fig. 4t. The dominating cellular forms in the certain Braak NFT stages are indicated with red marks. *Debris is accumulating in two clusters around two disintegrating cells.

Examined parameter	***'Braak stage' factor***		***'Dorso-ventral axis' factor***		***Interaction***	
	F/dF values	***p values***	***F/dF values***	***p values***	***F/dF values***	***p values***
Proportional distribution of AT8⁺ immunostaining volume, normalized for segment volume	$F_{(3,30)}=1.902 \times 10^{-13}$	*n.s.*	$F_{(1,30)}=39.75$	$p<0.0001$	$F_{(3,30)}=1.738$	*n.s.*
Proportional distribution of AT8⁺ immunostaining volume, normalized for TH⁺ cell number	$F_{(3,30)}=2.79 \times 10^{-14}$	*n.s.*	$F_{(1,30)}=22.94$	$p<0.0001$	$F_{(3,30)}=2.42$	*n.s.*
Proportional distribution AT8⁺ cell bodies, normalized for segment volume	$F_{(3,30)}=3.84 \times 10^{-14}$	*n.s.*	$F_{(1,30)}=84.16$	$p<0.0001$	$F_{(3,30)}=5.943$	$p<0.01$
Proportional distribution of AT8⁺ cell bodies normalized for TH⁺ cell number	$F_{(3,30)}=3.56 \times 10^{-14}$	*n.s.*	$F_{(1,30)}=43.94$	$p<0.0001$	$F_{(3,30)}=9.028$	$p<0.001$

Supplementary table 4 Proportional dorso-ventral distribution of $AT8^+$ immunostaining volume and $AT8^+$ cell body number in the LC core: summary of two-way ANOVA analyses. Statistically significant results are reported in red.

MOVIE LEGENDS

All movies have been compressed due to file size restrictions. Higher quality versions of all movies are available here: https://volume-imaging.com/tau-pathology-of-the-noradrenergic-human-locus-coeruleus-in-3d/high-resolution-versions-of-supplementary-videos-in-the-paper-of-gilvesy-et-al-acta-neuropath-2022/.

Video #1 LC core from a Braak 0 brain, TH volume immunostaining.

Segmented LC core, 2.42 μm x 2.42 μm x 2.50 μm voxel dimensions.

0:00 – 0:50 min.: Video demonstration of LC core cytoarchitecture.

0:50 – 1:10.: Demonstration of a small artery dorsally entering the LC core. The wall of the vessel is surrounded by densely packed TH^+ neurites.

Video #2 LC shell from a Braak 0 brain, TH volume immunostaining.

300 μm x 900 μm x 2700 μm 3D crop, focusing to the LC shell. 1.51 μm x 1.51 μm x 2.00 μm voxel dimensions.

LC shell exhibits low TH^+ cell density but a dense plexus of TH^+ processes, mainly dendrites. Ventrally from the sparse shell TH^+ neurons, long TH^+ axons of the dorsal noradrenergic bundle are shown.

Video #3 A4 from a Braak 0 brain, TH volume immunostaining.

Segmented A4, 1.51 μm x 1.51 μm x 2.00 μm voxel dimensions.

A4 consisted of sparse TH^+ cells with distinctly elongated somas. Their processes form a complex plexus around the mesenchephalic trigeminal tracts (*me5*). These processes are shown between 0:15 – 0:30. in details. *Me5* is segmented and represented by a white surface between 0:00 – 0:07.

Video #4 Tau cytoskeletal pathology in the LC of a Braak NFT stage 0 brain.

Representative 3D crop from the LC core. AT8 channel. 2.42 µm x 2.42 µm x 2.50 µm voxel dimensions.

Sparse AT8$^+$ cells with inhomogeneously or homogeneously filled somas and intact processes are dominating. AT8$^+$ axon of a tau-bearing neuron is followed until the end of the 3D crop in between 0:47 – 1:06.

Video #5 Tau cytoskeletal pathology in the LC of a Braak NFT stage 1 brain.

Representative 3D crop from the LC core. AT8 channel. 1.51 µm x 1.51 µm x 2.00 µm voxel dimensions.

Strongly filled cells with partially atrophic dendritic tree are dominating. However, several transition forms are noticed in this sample.

Video #6 Tau cytoskeletal pathology in the LC of a Braak NFT stage 3 brain.

Representative 3D crop from the LC core. AT8 channel. 1.51 µm x 1.51 µm x 2.00 µm voxel dimensions.

Strongly filled cells with severely atrophic processes are dominating. Zoom to examples of strongly filled cells with severely atrophic processes between 0:55 – 1:00.

Video #7 ***Tau cytoskeletal pathology in the LC of a Braak NFT stage 6 brain (Alzheimer's disease).***

Representative 3D crop from the LC core. AT8 channel. 1.51 µm x 1.51 µm x 2.00 µm voxel dimensions.

Abundant $AT8^+$ mature tangle-like cells are dominating. Zoom to a neighboring tau-bearing cell duo between 0:38 – 0:43.

Video #8 Dorso-ventral distribution of $AT8^+$ volume immunostaining in the LC.

Segmented LC core from a Braak NFT stage 3 brain. TH (gray) and AT8 (red) channels. 4.83 μm x 4.83 μm x 4.00 μm (downsampled) voxel dimensions.

0:00 – 0:06 min.: TH and AT8 channels are shown.

0:07 – 0:40 min.: only AT8 channels is shown.

0:40 – 1:02 min.: Red and gray surfaces indicate AT8 volume staining in the dorsal and ventral halves of the LC core, respectively. Blue surface covers the entire LC core volume. Note the more abundant AT8 volume staining in the dorsal half.

Video #9 Demonstration of dense cells.

3D crop from the LC core of a Braak NFT stage 2 brain. TH (gray) and AT8 (red) channels. 1.51 μm x 1.51 μm x 2.00 μm voxel dimensions.

0:00 – 0:05 min.: only TH channel, dense LC core cells are shown.

0:05 – 0:12 min: TH and AT8 channels are shown.

0:12 – 1:02 min.: only AT8 channel is shown; 0:40 – 0:45 zoom to a minigroup (formed by three cells with inhomogeneously filled soma and intact processes). 0:46 – 0:50: zoom to another minigroup, formed by (i) a cell with partially atrophic dendritic tree, (ii) a cell with severely atrophic processes and by (iii) cell with low intensity. 0:54 – 0:58: zoom to an $AT8^+$ cell duo, formed by two cells with partially atrophic dendritic tree.

Video #10 Dendrites of $AT8^+$ LC NA cells extend into the subependymal NA plexus already in Braak 0 stage.

3D crop from a Braak NFT stage 0 brain block: dorsal-medial parts of the LC core, part of A4 as well as the periventricular area are shown. TH (gray) and AT8 (red) channels. 1.51 μm x 1.51 μm x 2.00 μm voxel dimensions.

Note the dense subependymal NA plexus below the 4th ventricle with sparse $AT8^{+}$ processes.

0:00 – 0:06 min.: TH channel only.

0:06 – 1:10 min: TH and AT8 channels together.

CITED LITERATURE IN SUPPEMENTARY INFORMATION

1 Diggle P (1983) Statistical Analysis of Spatial Point Patterns. Academic Press, City

2 Tanaka N, Kanatani S, Kaczynska D, Fukumoto K, Louhivuori L, Mizutani T, Kopper O, Kronqvist P, Robertson S, Lindh Cet al (2020) Three-dimensional single-cell imaging for the analysis of RNA and protein expression in intact tumour biopsies. Nat Biomed Eng 4: 875-888 Doi 10.1038/s41551-020-0576-z
